# Microstructure and tribological behavior of Al–TiC composite strips fabricated by a multi-step densification method

**DOI:** 10.1038/s41598-024-70560-x

**Published:** 2024-09-05

**Authors:** Mohamed I. A. Habba, Waheed S. Barakat, Sarah A. Elnekhaily, F. S. Hamid

**Affiliations:** 1 Mechanical Department, Faculty of Technology and Education, Suez University, Suez, 43221 Egypt; 2 Metallurgical and Materials Engineering Department, Faculty of Petroleum and Mining Engineering, Suez University, Suez, 43221 Egypt

**Keywords:** Multi-step densification, Al–TiC composites, Automotive applications, Mechanical properties, Tribological properties, Engineering, Materials science

## Abstract

This study aims to enhance the tribological properties of automotive applications by examining the effects of TiC content on the microstructure, mechanical properties, and wear behavior. This study investigates the production of Al–TiC composite strips using a novel multi-step densification process combining mechanical alloying and hot rolling with TiC concentrations ranging from 0 to 12 vol%. The novelty of this work lies in its comprehensive approach to developing and analyzing Al–TiC composite strips using a multistep densification method. This study integrates microstructural analysis, mechanical property evaluation, and detailed tribological behavior assessment under different wear loads (5–25 N). A key innovation is the application of the Abbott Firestone method to analyze worn surfaces, providing insights into optimal wear conditions. The study reveals that increasing the TiC content to 12 vol% significantly improves densification, hardness (up to 268.8% increase), and wear resistance (up to 95% improvement at a 5N load). Dry ball-on-flat sliding wear tests at loads of 5–25N demonstrate that TiC particles hindered complete delamination wear in the composite strips. The Abbott Firestone method analysis of worn surfaces indicated an optimal exploitation zone in the Al-6 vol% TiC composite at both low and high wear loads. This comprehensive approach provides valuable insights into optimizing Al–TiC composites for enhanced performance in automotive components that require improved wear resistance.

## Introduction

Aluminum metal matrix composites (AMMCs) are advanced materials that combine the benefits of aluminum (Al) as a matrix with other materials, such as particles or fibers^1,2^, which act as reinforcements. This results in a stronger, harder, and more heat-resistant material than pure Al^3,4^. AMMCs are used in various applications, including aerospace, automotive, and defense industries. Overall, AMMCs are valuable materials for a wide range of applications because of their unique properties and ability to improve the performance of Al-based products^5,6^. Ceramic particulate reinforcements are an excellent choice for forming AMMCs, owing to their numerous advantages. One of the main advantages is their high strength and stiffness, which allows for the design of lightweight, high-performance AMMCs^7^. They also exhibit excellent wear resistance, making them suitable for use in abrasive environments^8,9^. Ceramic particles that produce advanced multifunctional ceramic materials (AMMCs) include TiB_2_^10^, Al_2_O_3_^11^, SiC^12^, TiC^13^, B_4_C^14^, and ZrO_2_^15^. TiC particles have several advantages, including excellent wear resistance and hardness, which make them suitable for applications that require high wear and abrasion resistance^16,17^. Furthermore, TiC has a low thermal expansion coefficient, which makes it compatible with Al and prevents cracking and delamination during thermal cycling^18^. Several manufacturing techniques can be used to produce AMMCs, such as powder metallurgy (PM)^2^, PM extrusion^19^, stir casting-rolling^20^, stir casting-extrusion^21^, accumulative roll bonding^22^, and spray co-deposition followed by extrusion^23^. PM involves mixing Al powder with reinforcement particles, compressing the mixture under high pressure, and sintering at a suitable temperature. This results in a more uniform distribution of reinforcement particles within the Al matrix^24^ than liquid-state processing methods, such as stir casting. Compared to composites produced by conventional casting techniques, the improved particle distribution from powder metallurgy can enhance strength, hardness, and wear resistance. Furthermore, mechanical alloying of the composite mixture involves high-energy ball milling to mix and mill the powders of the composite components^2^. Thus, a nanocomposite mixture can be produced^25,26^. Previous research efforts have primarily concentrated on Al–TiC composites created through PM methods. Azimi et al.^41^ investigated Al7075-TiC nanocomposites produced by mechanical alloying and hot pressing, finding that 5 wt% nano-TiC optimally improved hardness and wear resistance, with a maximum tensile strength of 725 MPa achieved under hot pressing conditions. Jeyasimman et al.^42^ studied Al 6061-TiC nanocomposites (1, 1.5, 2 wt% TiC) synthesized via mechanical alloying, focusing on consolidation and mechanical behavior. Albert et al.^47^ prepared and characterized Al-5 and 10 wt% TiC composites using powder metallurgy technique. Alam et al.^59^ developed an Artificial Neural Network model to predict the effect of milling time and TiC content on crystallite size and lattice strain of Al7075- 4 wt% TiC composite made by powder metallurgy, achieving good agreement between predicted and experimental results. Cabeza et al.^60^ examined the effects of high-energy ball milling on 6005A Al alloy matrix composites reinforced with nano-sized TiC particles (1.5, 3, and 6 vol%). Salur et al.^61^ improved the mechanical properties of AA7075 Al alloy composites reinforced with 5 wt% nano-sized TiC particles using ball milling and hot pressing, achieving a threefold increase in hardness and 40% higher ultimate tensile strength compared to the original alloy. Hamid et al.^62^ investigated the morphology and mechanical properties of Al–TiC nanocomposites reinforced with 10–20 wt% TiC and processed by ball milling.

In general, applying a rolling regime after the PM method results in a more uniform distribution of particulate reinforcements, increases material densification, and enhances mechanical properties such as strength^27–30^. Yuan et al.^31^ studied the combined effect of powder metallurgy and hot rolling to produce Al composites reinforced with carbon nanotubes (CNTs). They found that this hybrid method improved the mechanical properties of the composite by increasing the distribution homogeneity of CNTs. The rolling process has several advantages when processing AMMCs synthesized using the PM process, such as achieving a high degree of homogeneity in rolled AMMCs^31,32^. This is because the rolling process applies high pressure to the synthesized AMMCs, resulting in a more uniform distribution of particles^27^. Additionally, the rolling process can produce a higher density of rolled AMMC materials, thereby improving the mechanical properties of the final AMMCs^33^. Based on the literature, a few previous studies have focused on exploring the effect of the rolling process on metal-based composites synthesized using the PM route. In addition, no investigations have examined the multistep densification technique of PM and the hot-rolling process to produce Al–TiC composite strips. The hot-rolled composite strips are expected to attain a relatively uniform distribution of TiC ceramic particles within the Al matrix, thereby improving the mechanical and tribological properties of the rolled composites. Table 1 summarizes previous studies that have focused on multi-step techniques to produce metal-based composites. More extensive and intensive studies should be conducted to investigate the effect of the multi-step densification method (powder metallurgy followed by hot rolling) on the production of metal-based composites. This study aims to produce Al–TiC composite strips using a multi-step densification method and investigate their densification, hardness, wear resistance, and associated microstructural characteristics. XRD analysis, density, and hardness of the hot-rolled strips were investigated. The tribological performance of the produced matrix (wear rate, FE-SEM of worn surfaces, wear tracks, and debris analysis) and their composites were investigated under different wear loading values ranging from 5 to 25N to detect the dominant wear mechanisms. In addition, the worn surfaces of the wear-tested samples were treated using the Abbott Firestone method to identify the optimal conditions for wear applications, and the surface roughness profiles were examined to achieve the desired outcome.

**Table 1:** Previous studies related to multi-step techniques for producing metal-based composites.

No	Matrix	Reinforcement	Manufacture technique	Major mentions	Refs
		Type/Concentration			
	Al	Al_2_O_3_/2, 4, 6, 10 wt%	Mechanical milling, hot pressing, and hot rolling	Increasing the tensile and hardness properties and improving the distribution of Al_2_O_3_	^32^
	Al	CNTs/1.5 wt%	PM and hot rolling	Improving the density, hardness, and tensile strength with a reduction in ductility	^31^
	Al	SiC/7 vol%	Mechanical milling, cold compaction, sintering, hot rolling	Uniform distribution, grain refining, increasing the strain hardening	^29^
	Cu	Reduced graphene oxide/2.47 and 3,27 vol%	Mechanical milling, hot pressing, and hot rolling	Uniform distribution and improving the tensile strength with a reduction in ductility	^63^
	Ti	–	Direct powder rolling	Improving corrosion resistance and density properties with a reduction in the porosity	^64^

## Experimental methods

**Fig. 1:**
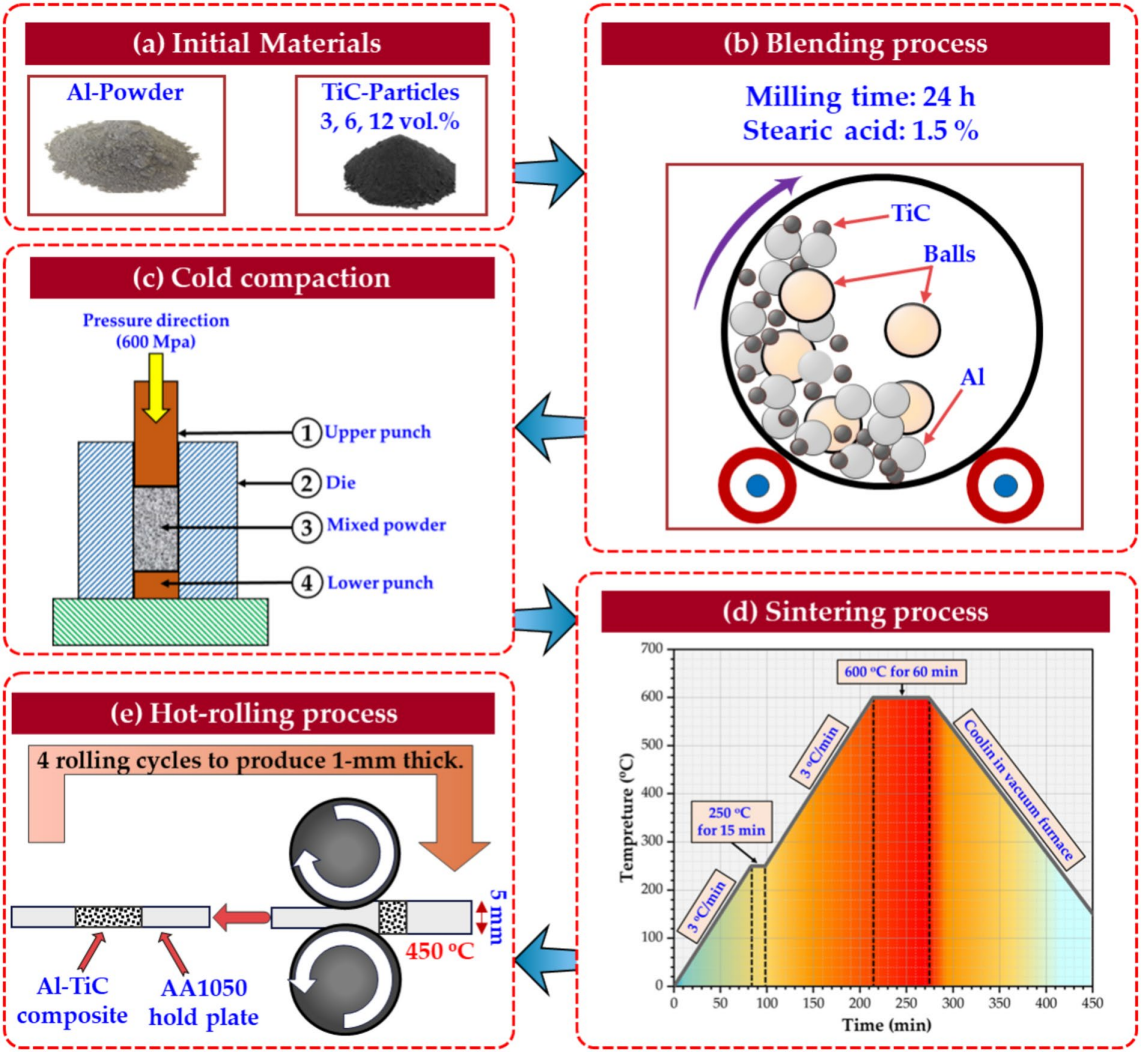
Schematic drawing of the procedure for the fabrication of Al–TiC composite strips.

The experimental procedure for producing Al–TiC composite strips using the hot-rolling technique was conducted in four steps: (1) blending of the selected powders, (2) cold compaction of the blended mixture, (3) sintering of the compacted composites to consolidate the Al–TiC composite, and (4) hot rolling of the consolidated Al–TiC composites to fabricate the targeted composite strips. Figure 1 shows a schematic of the experimental sequence used in this study.

### Initial materials

To fabricate the Al–TiC composite strips, high-purity Al powder with a purity of 99.98% was used as the matrix according to the supplier (Egyptian Aluminum Company, Egypt). TiC particles with 99.70% purity were obtained from Alpha Chemicals (USA) (Fig. 1a).

### Synthesis of Al/TiC composites

Three Al-based composite strips were fabricated with different quantities of TiC (3, 6, and 12 vol%) in addition to a pure Al matrix with 0% TiC. For each TiC content under an argon atmosphere, the Al matrix and TiC powders were mixed using hard chrome balls and milled for 24 h at a 110-rpm rotation speed to obtain a homogeneous distribution of the powders^2^. The hard chrome grinding ball weighed 35:1, compared with the blended powders. Furthermore, stearic acid 1.5 wt% is utilized to control the milling process of Al–TiC composite powder, as shown in Fig. 1b. Subsequently, the mixed Al–TiC composite was heated in a tube furnace at 400 °C for 30 min in an argon gas environment to eliminate stearic acid before cold compaction. The blended Al–TiC composite powders were subjected to cold compaction using a universal testing machine (INSTRON/4208–002/350 KN capacity, Norwood, MA, US) at a pressure of 600 MPa, as shown in Fig. 1c. The cold-compacted composites were sintered at 600 °C for 60 min in a vacuum furnace (Zhengzhou Brother Furnace Co. Ltd., Zhengzhou, China). Figure 1d illustrates the sintering cycles of the cold-compacted composites.

### Hot-rolling of Al–TiC composites

The sintered Al–TiC cold specimens were encapsulated in an AA1050 hold plate to facilitate rolling of the composite specimens, as illustrated in Fig. 1e. The hot-rolling process of the cold-compacted samples was conducted using a laboratory rolling machine (Robertson rolling mill, Mogliano Veneto, Italy) with a 20-ton loading capacity. Al–TiC composites encapsulated in AA1050 plates were soaked in incombustible grease to reduce friction during the rolling process. Four rolling cycles were conducted to attain a 1 mm thickness of Al–TiC composite strips. The specimens were heated to 450 °C for 5 min for each rolling cycle using a 4.5 m/min rolling speed.

### Characterization of rolled Al–TiC composite strips

Phase analysis of the hot-rolled Al–TiC composite strips was performed using (XRD) analysis. Diffraction patterns were recorded using an X-ray system ( Siemens/Bruker D5000 XRD system, KS Analytical Systems Company, Texas, US) radiations of K-alpha (1.54056 Å) from a copper X-ray tube in 2-Theta ranging from 20° to 80° in 0.1°steps.

The crystallite size was calculated from the XRD data using the Williamson-Hall relation according to Eq. (1).

1 $$D = \frac{K\lambda }{{\beta \times cos\theta }}$$

where D is the mean crystallite size, K is the shape factor (typically 0.94), λ is the X-ray wavelength (1.54178 nm for Cu Kα radiation), β is the full width at half maximum (FWHM) of the diffraction peak in radians, and θ is the Bragg angle. The dislocation density can be estimated using Eq. (2):

2 $$dislocation \;density = \left( {1/D^{2} } \right)$$

where, *D* is the calculated crystalline size.

The microstructures of the hot-rolled Al-matrix and their corresponding Al–TiC composites were examined using Field-Emission Scanning Electron Microscopy (Model: QUANTA FEG 250, FEI Company, Hillsboro, USA) equipped with advanced Energy-Dispersive X-ray Spectroscopy (EDS) to specimens that were cut in the rolling direction (RD) as well as in the transverse direction (TD). The densities of the hot-rolled strips were measured according to Archimedes’ principle. Distilled water was used as the immersion liquid to provide an electronic balance ( BA-E Series, Lixia District, Jinan, Shandong, China) with an accuracy of 1 × 10^−4^ g. The hardness of the strips was measured using a Vickers hardness tester (FM-ARS 9000, Spectrographic Limited, Leeds, UK) at an applied load of 300 g and a loading time of 15 s. For each composite strip, at least eight reads were randomly measured on the top surface of the hot-rolled strips, and the average value was determined.

**Fig. 2:**
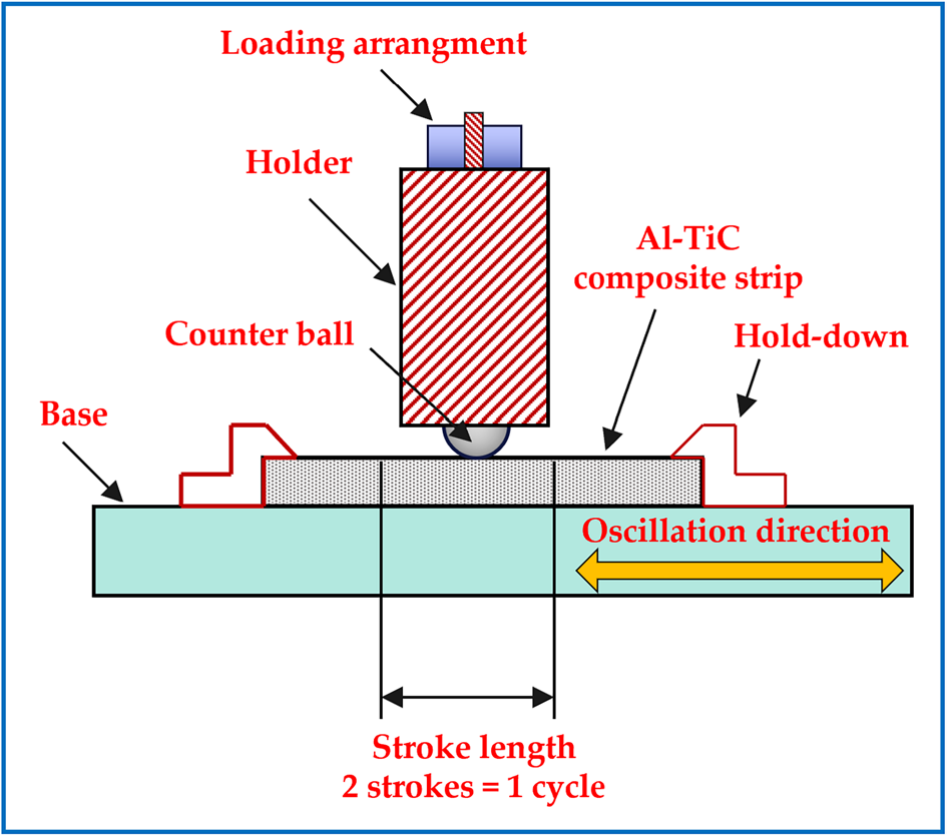
Illustration of the reciprocating ball-on-flat sliding wear mechanism.

The sliding wear on the Al–TiC composite strips was maintained using a ball-on-flat setup under unlubricated wear testing at room temperature (according to ASTM G133-02) on an EGY-TEF-M1 wear-tester laboratory machine (Suez University, Suez, Egypt). The counter body (ball) used in the wear tests was made of AISI 52,100 chrome steel, with a hardness of 63 HRC, diameter of 10 mm, and surface roughness of 0.05 μm. The linearly reciprocating ball-on-flat sliding wear method involves two specimens: a flat specimen (Al–TiC composite strips) and a spherically ended (herein called the “ball”) specimen (counterbody) that slides against the flat specimen. These specimens moved relative to each other in a linear, back-and-forth sliding motion under the prescribed set of conditions, as listed in Table 2. This wear-test method applies a load vertically downward through the ball counter-body against horizontally mounted Al–TiC composite strips, as shown in Fig. 2. The dimensional changes in the flat specimens were used to calculate the wear volumes and rates. The volumetric wear rate was calculated using the formula shown in Eq. (3):

3 $$W = \frac{m}{\rho \times S}$$

where W is the volumetric wear rate (mm^3^/m), V the mass loss due to wear (mm^3^), ρ is the material’s density (g/mm^3^), and S is the total sliding distance (m). The wear mechanisms were suggested according to the morphologies of the worn surfaces and debris examination using FE-SEM supported by EDS. In addition, the size distribution of the debris collected during the wear test was analyzed using ImageJ software.

**Table 2:** Linear reciprocating ball-on-flat sliding wear conditions.

Pin tip radius	Normal force	Stroke length	Number of cycles
5 mm	Ranges from 5 to 25 N	10 mm	2500

Test duration	Total distance	Ambient temperature	Lubrication
16 min 40 s	100 m	22 ± 2 °C	None applied

## Results and discussions

### Characterization of initial materials

**Fig. 3:**
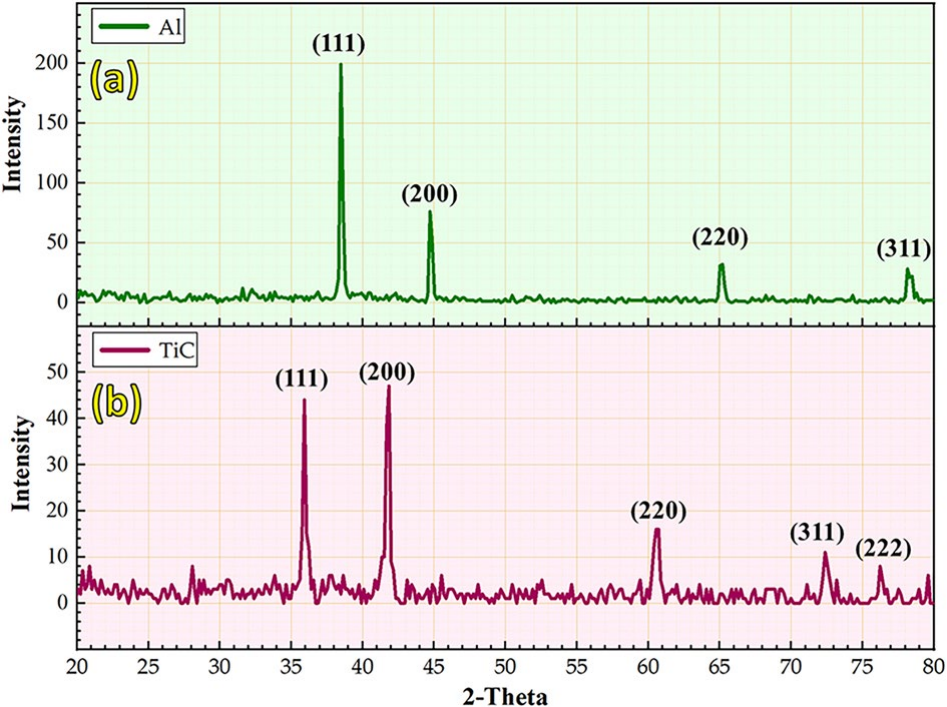
XRD patterns of (**a**) Al and (**b**) TiC as-received powder.

**Fig. 4:**
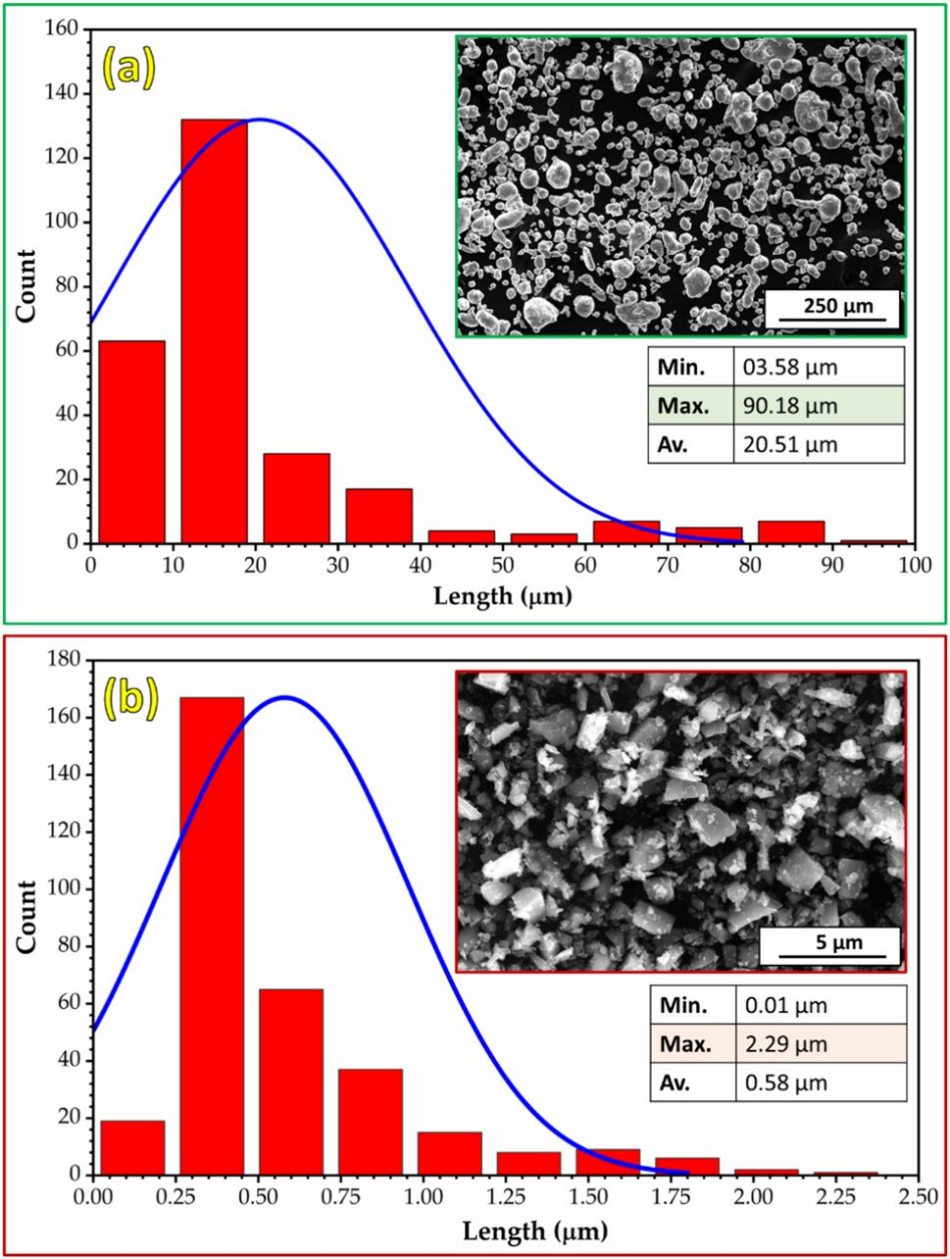
FE-SEM images and particle size analysis of (**a**) Al and (**b**) TiC as-received particles.

The phase composition and morphology with particle size analysis of the as-received Al and TiC powders were characterized using XRD (Fig. 3) and FE-SEM (Fig. 4), respectively. The XRD patterns of the Al and TiC powders are shown in Fig. 3. The Al powder XRD pattern (Fig. 3a) exhibited four main peaks at 2θ angles of 38.5° (111), 44.7° (200), 65.2° (220), and 78.2° (311), corresponding to the face-centered cubic structure of Al. The TiC powder XRD pattern (Fig. 3b) shows five prominent peaks at 2θ angles of 35.9° (111), 47.0° (200), 60.5° (220), 72.4° (311), and 76.3° (222), confirming the presence of the TiC phase. FE-SEM analysis revealed that the Al powder particles had an approximately spherical morphology with a wide size distribution ranging from 3.58 to 90.18 μm and an average size of 20.51 μm (Fig. 4a). In contrast, the TiC powder particles exhibited an irregular, sharp-edged morphology with a narrower size range of 0.01 to 2.29 μm and an average size of 0.58 μm (Fig. 4b).

### Characterization of hot-rolled strips

#### Microstructural analysis

**Fig. 5:**
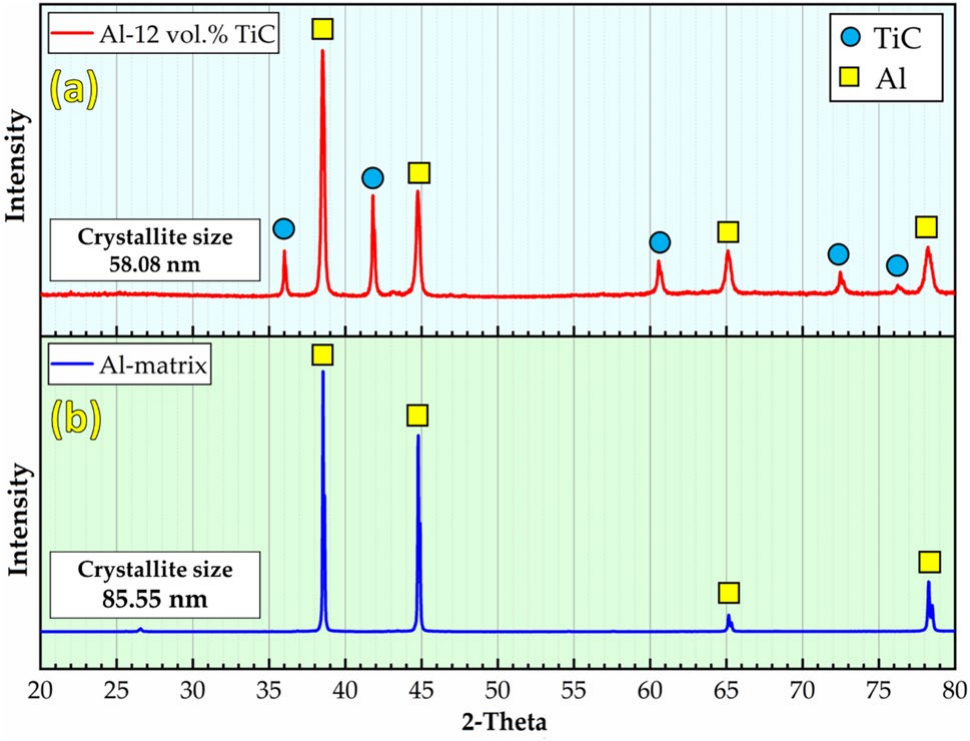
XRD patterns of hot-rolled strips of (**a**) Al-12 vol% TiC composite and (**b**) Al-matrix.

The XRD patterns of the TiC-free sample and the Al with the highest TiC content of 12 vol% after the hot-rolling process are illustrated in Fig. 5. The main peaks of Al and TiC were detected without any formation of new intermetallic phases between the Al matrix and TiC particles during the hot-rolling process. In previous studies^34,35^, intermetallic Ti_3_Al and AI_4_C_3_ were observed during the production of Al–TiC composites when the temperature reached ~ 640 °C. The formation of Al₄C₃ during the production of the Al–TiC composites can be explained by the interaction between Al and carbon at elevated temperatures. The specific reaction that leads to the formation of Al₄C₃ is as shown in Eq. (4).

4 $$4Al + 3C \to Al_{4} C_{3}$$

In the context of Al–TiC composites, the presence of carbon is attributed to TiC particles. When the composite is subjected to high temperatures, such as during sintering or hot rolling, the Al matrix can react with the carbon from the TiC particles to form Al₄C₃. This reaction is thermodynamically favorable at temperatures around 640°C and above, as indicated by Gibbs free energy calculations for the reaction^36^. In the present study, neither Ti_3_Al or AI_4_C_3_ intermetallics were not detected, because the maximum temperatures encountered in the sintering and hot-rolling processes were lower than the aforementioned temperature. Notably, this is a significant advantage of solid-state techniques for producing composites over liquid techniques, such as casting^37^. For the XRD of Al-12 vol%, it can be observed that the intensity of the Al peaks decreased, and their broadening increased compared to the rolled Al-matrix; this could be attributed to the reduction in the concentration of the Al-matrix in the rolled composite by adding TiC particles. Furthermore, the broadening of the peaks indicates a reduction in the crystallite size during hot rolling. The crystallite size and dislocation density of the hot-rolled materials were calculated according to the Williamson-Hall relation, as shown in Fig. 5. The crystallite size and dislocation density are 58.08 nm and 3.33 × 10^–4^, respectively, for the Al-12 vol% TiC composites and 85.55 nm and 1.38 × 10^–4^, for the Al-matrix rolled materials, as presented in Fig. 5a,b. The reduction in the crystallite size of the Al-12 vol% TiC rolled composites compared to the rolled Al-matrix can be attributed to several factors; the presence of TiC particles acts as obstacles to grain growth during the hot rolling process. These particles pin the grain boundaries, limiting grain coarsening and promoting the formation of finer grains^31,38^. Furthermore, the hot rolling process, combined with the presence of hard TiC particles, induces severe plastic deformation in the Al matrix. This deformation can lead to grain fragmentation and the formation of subgrains, effectively reducing the crystallite size^39,40^. In addition, TiC particles can act as pinning points for dislocations, thereby preventing their movement and annihilation. This can lead to the formation of dislocation cell structures with low-angle grain boundaries, further contributing to the reduction in crystallite size^7,11^. Azimi et al.^41^ reported that the addition of TiC nanoparticles to an Al7075 matrix led to increased dislocation density and grain refinement, which contributed to improved mechanical properties. Jeyasimman et al.^42^ observed that incorporating TiC particles into an Al 6061 matrix resulted in higher dislocation density and enhanced hardness. Cabeza et al.^43^ found that TiC particle reinforcement in an Al 6005A alloy increased dislocation density and improved strength. Lin et al.^44^ noted that TiC particles in an Al matrix act as obstacles to dislocation motion, leading to increased dislocation density and improved mechanical properties. Nemati et al.^45^ reported that TiC nanoparticles in an Al-Cu alloy increased dislocation density and enhanced hardness.

**Fig. 6:**
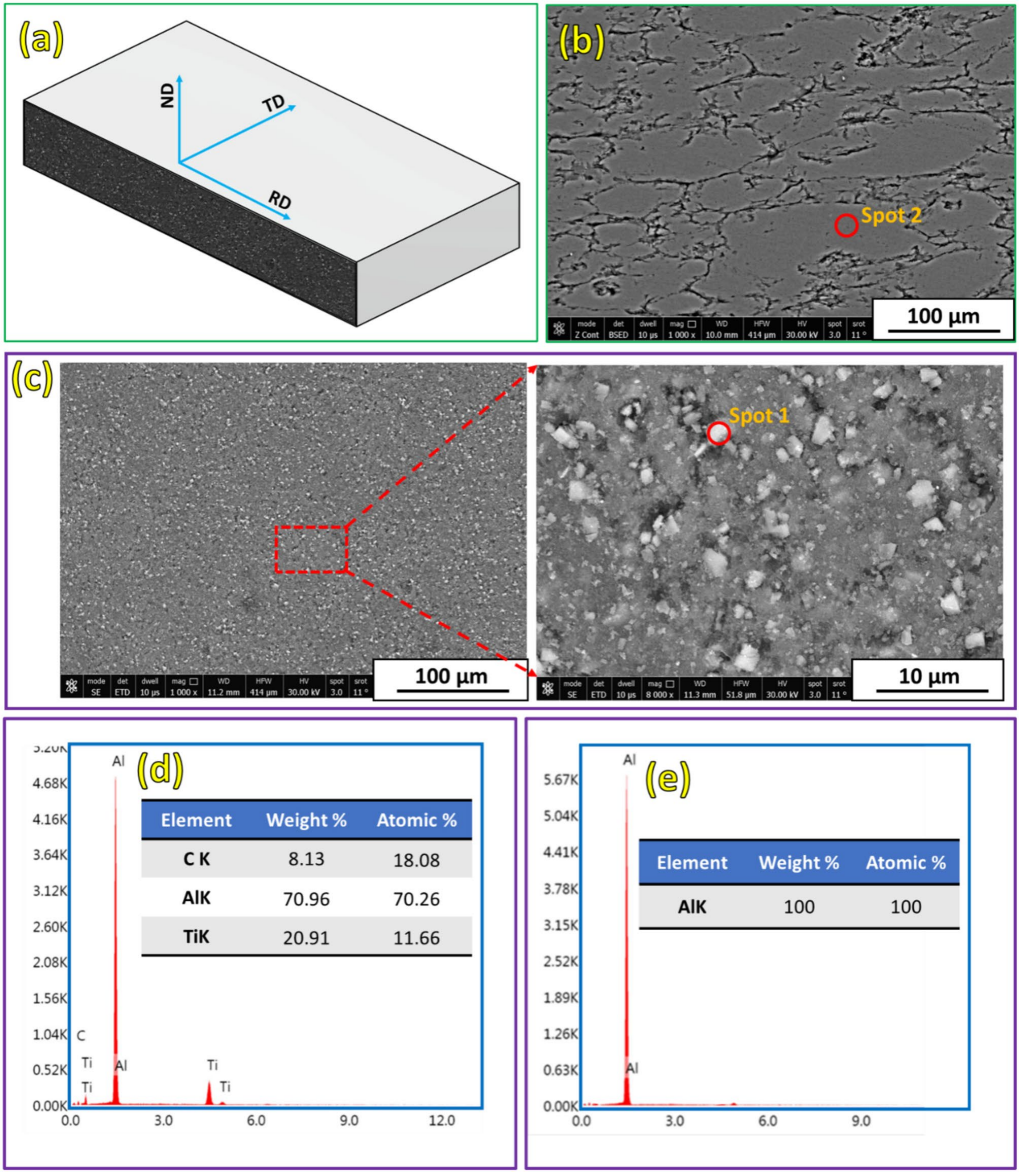
(**a**) Illustration of the microstructure location and FE-SEM images of hot-rolled strips of (**b**) Al matrix, (**c**) Al-12 vol% TiC. (**d**) and (**e**) EDS analysis of the spot 1 in (**b**) and spot 2 in (**c**), respectively.

Figure 6a shows a schematic illustration of the cross-section of the hot-rolled samples, indicating the rolling direction (RD) and the area examined by FE-SEM. FE-SEM micrographs of the hot-rolled Al matrix and its composite strips were then examined across their thicknesses along the rolling direction, as shown in Fig. 6b,c. Furthermore, Fig. 6d,e show the EDS analysis of spot-1 and spot-2 in Fig. 6**,** respectively. This overview of the microstructure analysis demonstrates the efficiency of the hot-rolling process applied to obtain a macro void-free material under compressive stress. The gray phase represents the Al matrix (Fig. 6e; spot-2), whereas the white scattered spots indicate the TiC phase (Fig. 6d; spot-1). For the hot-rolled Al–TiC composite, the high-magnification image in Fig. 6c confirms that the TiC particles were more or less uniformly distributed throughout the Al matrix with few agglomeration sites. The microstructure of the hot-rolled materials revealed that the hot-rolling step of the composites produced by the PM process positively affected the developed microstructure because it caused grain refinement of the rolled materials and enabled the fragmentation and redispersion of the particles in the matrix owing to the thermomechanical effect. Thus, the applied experimental procedure is recommended for producing Al-based composites with a good distribution of ceramic particles.

**Fig. 7:**
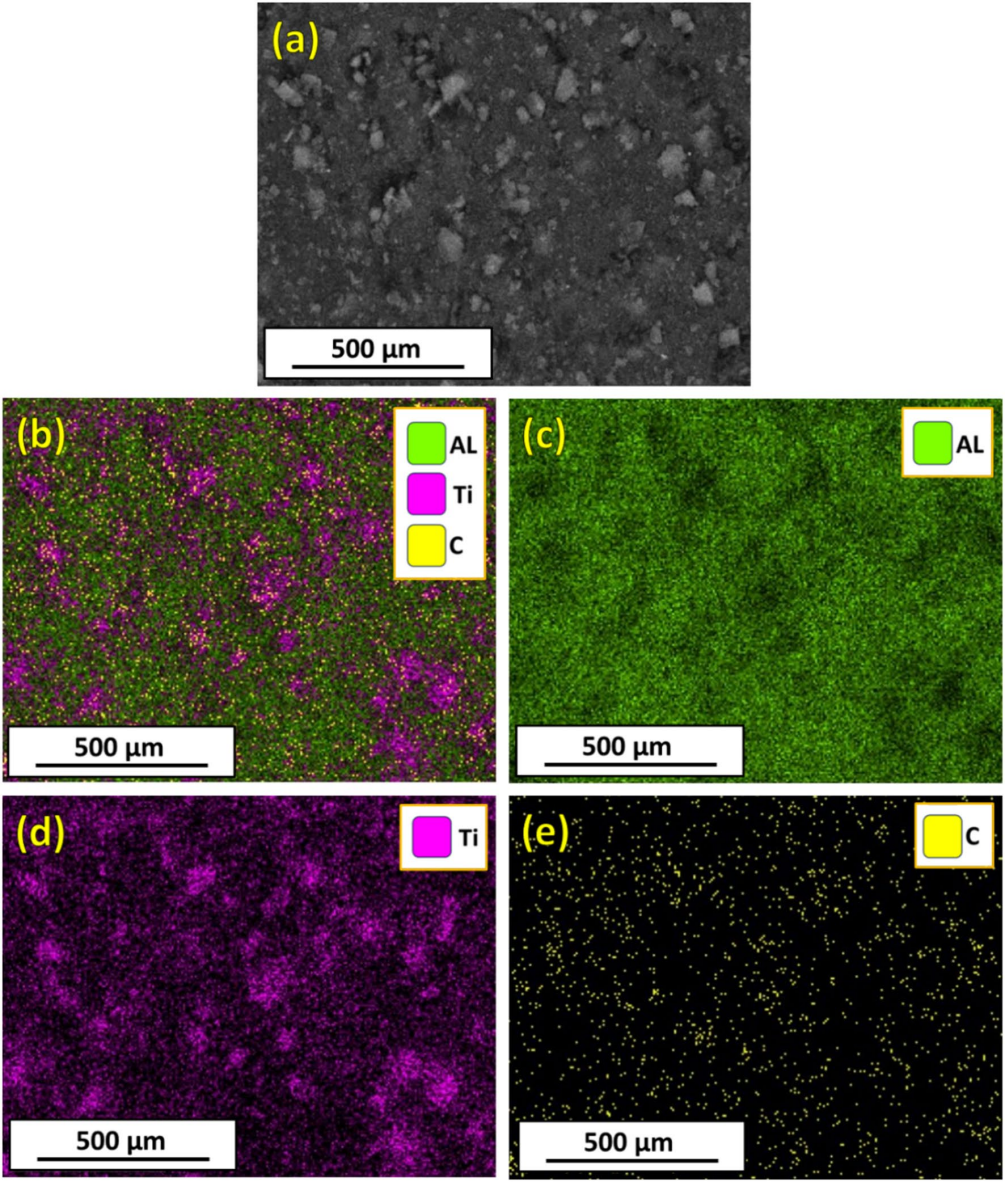
Colored map of the Al-12% vol% TiC hot rolled composite strip.

**Fig. 8:**
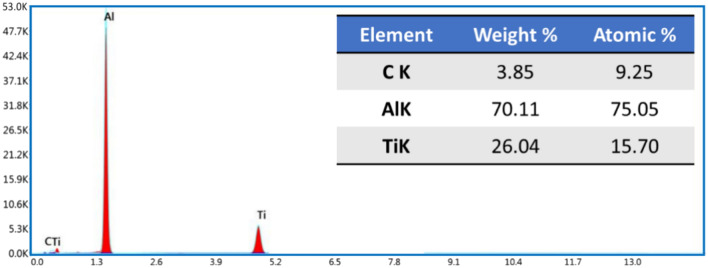
EDS analysis of the Al-12% vol% TiC hot rolled composite strip.

Elemental mapping with EDS analysis was conducted to determine the composition of the hot-rolled composite strips and the distribution of the reinforcing TiC particles in the Al matrix. Figure 7a shows the FE-SEM image, and Fig. 7**-b** shows the combined elemental colored map of the Al-12 vol% TiC composite strip. It is observed that the elemental-colored maps confirm the composition of the hot-rolled Al–TiC composite strip in terms of Al (green dots; Fig. 7c), Ti (red dots; Fig. 7d), and C (yellow dots; Fig. 7e). It should also be noted that there was a uniform distribution of TiC particles without appreciable segregation. In addition, The EDS analysis in Fig. 8 also proves that the elemental compositions and their weight percentages are as expected; thus, no foreign elements were accidentally added (Fig. 8).

#### Density and hardness properties of hot-rolled composites

**Fig. 9:**
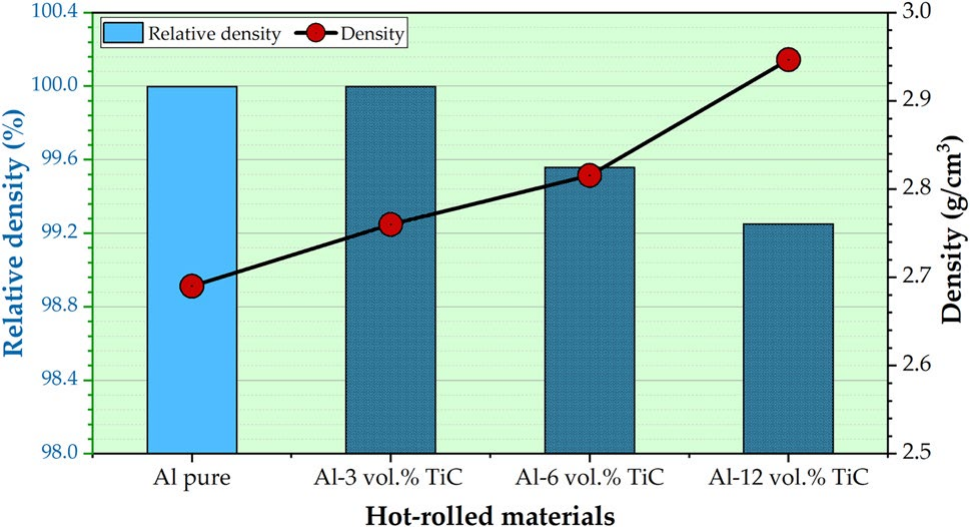
The bulk and relative densities of the hot-rolled pure Al and its composites reinforced with 3, 6, and 12 vol% of TiC particles.

The hot-rolling process of the Al matrix and composites of the Al–TiC strips can significantly affect the densification of the hot-rolled strips. Figure 9 shows the density and relative density of the hot-rolled Al strip and its composites reinforced with different TiC concentrations (3, 6, and 12 vol%). It can be observed that the densities of the Al–TiC strips increased with increasing TiC content from 0 to 12 vol%. In addition, the relative density shows that rolled Al and Al-3 vol% TiC strips have full densification (without any porosity), and the Al–TiC composite strips reinforced with 6 and 12 vol% TiC have near-full densifications of 99.559% (0.441% porosity) and 99.251% (0.749% porosity), respectively. These higher densifications could be attributed to (i) the applied pressure during the initial process of cold pressing (600 MPa; Fig. 1c), (ii) the composite entry hot rolling temperature (450 °C; Fig. 1e), which is near the Al dynamic recrystallization temperature, and (iii) the total reduction during the rolling process, leading to plastic deformation and redispersion of the ceramic particles. Several studies have demonstrated that the hot rolling of metal matrix composites leads to significant microstructural changes. For example, Li et al.^30^ observed that hot rolling of AA6061-SiC composites resulted in a more uniform distribution of SiC particles within the AA6061 matrix and increased densification. Similarly, Yuan et al.^31^ found that hot rolling of Al-CNT composites improved the homogeneity of distribution for CNTs. Kumar et al.^46^ noted that hot rolling of Al6061-ZrB2 composites caused the grains to elongate in the direction of the rolling process. The plastic deformation process leads to an increase in the densities of the produced composites because it results in the closure of intergranular voids and enhances the packing of atoms within the material. Additionally, the application of high pressure during rolling creates a more uniform distribution of particles in the composite material^46^. While grain boundaries are not clearly visible in the SEM images (Fig. 6c) of the Al-12vol% TiC composite strip, the Al matrix without TiC reinforcement (Fig. 6b) shows a more uniform appearance, suggesting potential grain elongation in the rolling direction. In contrast, the Al-12vol% TiC composite (Fig. 6c) microstructure likely results from a combination of constrained matrix deformation due to TiC particles and possible dynamic recrystallization during hot rolling. This process increases the overall density owing to the closure of intergranular voids and improved packing of atoms within the material. Accordingly, the applied rolling loads significantly reduced the void content, increased the packing density of atoms within the material, promoted the uniform dispersion of TiC particles within the Al matrix, and eventually enhanced the density of the rolled Al–TiC composites. Furthermore, the density of 2.93 g/cm^3^ compared to the Al matrix of 2.7 g/cm^3^; hence, the inclusion of TiC aids in increasing the density of the rolled composite strips^41,47^. The overall influence of TiC addition, cold compaction, and the hot-rolling process is expected to contribute to the improved properties of the rolled Al–TiC composite strips, including strength, hardness, and wear properties.

**Fig. 10:**
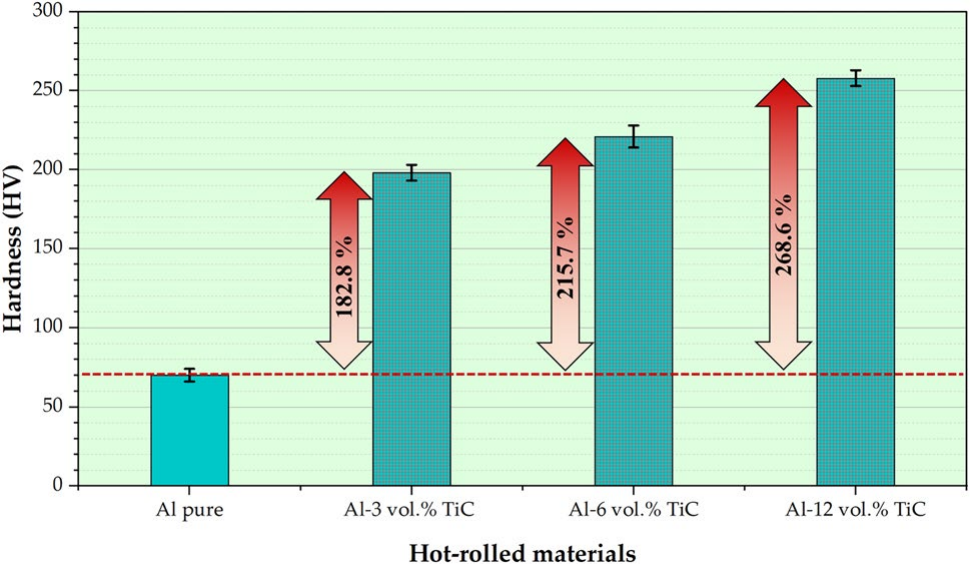
Vickers hardness measurements of hot-rolled Al strips and Al–TiC composite strips.

Hardness measurements are crucial in various industries. They provided valuable insights into the resistance of materials to deformation, wear, and strength. The hardness of the hot-rolled Al matrix and its composites on the surface of the rolled strips was measured, as illustrated in Fig. 10. Figure 10 shows that the rolled Al matrix hardness improved after applying the hot-rolling process for all reinforcement concentrations. The lowest hardness of 70 ± 4 HV was detected in the rolled Al matrix, which improved to the highest value of 258 ± 5 HV for the rolled Al-12 vol% TiC composite strip with an increase of ~ 268.6%, as presented in Fig. 10. Habba et al.^2^ synthesized Al–TiC composites using a powder metallurgy technique (milling process, cold compaction, and sintering) with 0, 3, 6, and 12 vol% of TiC under various milling times (6, 12, 24, and 48 h). They reported that the hardness of mechanically alloyed Al–TiC was improved compared to that of the Al matrix, and the hardness results were in the range of 33–137 HV for the Al matrix and Al-12 vol% TiC samples, respectively. In the present study, the hardness of the hot-rolled Al-matrix strip (70 HV) was higher than that previously reported^2^. Hot rolling is a thermomechanical process in which a rolled sheet is subjected to relatively high temperatures and compressive forces^29,39^. The sheet underwent plastic deformation under rolling pressure, which refined the grain structure and aligned the crystals, resulting in a more uniform and fine-grained microstructure^27,48^. The combined effect of temperature and plastic deformation enhances the dislocation density within the material, hinders the movement of dislocations, and increases the hardness of the hot-rolled Al-matrix strip compared to that determined in^2^. It is noteworthy that the existence of TiC ceramic particles in the Al matrix during the hot-rolling process contributed to the improvement in the hardness of the rolled Al matrix strip by approximately 182.8%, 215.7%, and 268,8% for the Al–TiC composite strips reinforced with 3, 6, and 12 vol%, respectively, Fig. 10. During the incorporation of TiC ceramic particles into the Al matrix, TiC particles, which are known for their high hardness and wear resistance, act as reinforcing agents^2^. During hot rolling, the particles were uniformly dispersed within the Al matrix (Figs. 6 and 7), promoting grain refinement through dynamic recrystallization and hindering grain growth. This refined microstructure increases the hardness owing to the presence of uniformly distributed TiC ceramic particles and the associated strengthening mechanisms, such as solid-solution strengthening, precipitation hardening, and grain boundary strengthening^49,50^. Ceramic particles also impede dislocation motion, enhance dislocation density, and increase strain hardening^31,46^.

The proper bonding between the TiC phase and Al matrix is evidenced by several key observations from this study. The FESEM images and EDS elemental maps (Figs. 6 and 7) reveal a homogeneous distribution of TiC particles throughout the Al matrix, indicating the absence of large agglomerations or clear phase separation. High-magnification FESEM images (Fig. 6c) show no visible voids or cracks around the TiC particles, suggesting good contact between the particles and the matrix. The density test results (Fig. 9) demonstrate a high relative density of up to 99.28% for the composite containing 12 vol% TiC, indicating very low porosity and good bonding between the composite components. Furthermore, the significant improvement in hardness (Fig. 10) with increasing TiC content supports the existence of effective bonding, as poor interfacial adhesion would typically lead to deterioration of hardness. These combined observations strongly suggest proper bonding between the TiC reinforcement and the Al matrix in the fabricated composites.

#### Tribological behavior

The wear rate, worn surfaces, wear tracks, and debris collected from the hot-rolled Al matrix and Al–TiC composite strips were investigated using ball-on-flat sliding wear tests. The wear performance and various wear mechanisms were verified under different wear loadings, along with the effects of hot rolling and TiC particles.

*Wear performance*

**Fig. 11:**
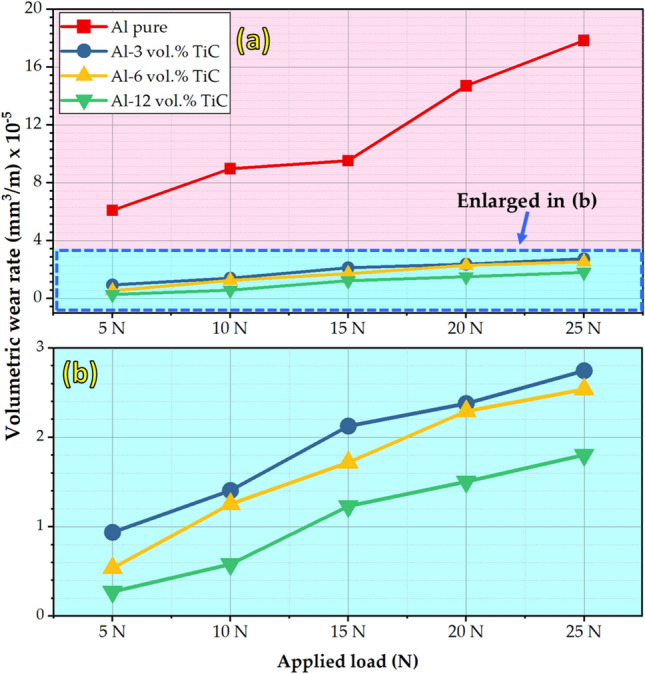
(**a**) Volumetric wear rate behavior of hot-rolled Al-Al matrix and–TiC composite strips using reciprocating ball-on-flat sliding wear mechanism at different wear loads of 5, 10, 15, 20, and 25 N. (**b**) Enlarged selected area in (**a**).

**Fig. 12:**
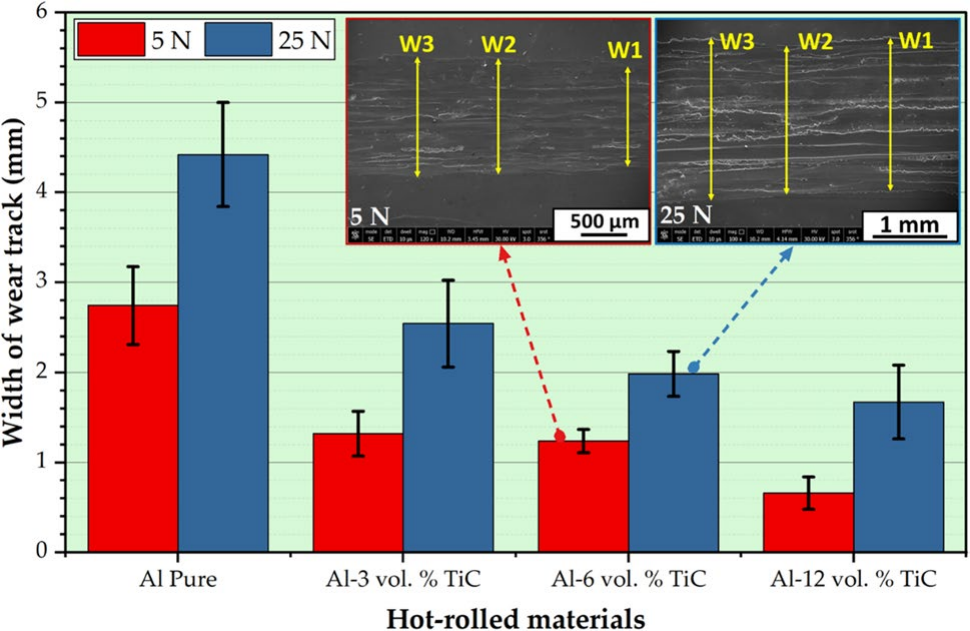
Wear track width of the hot-rolled Al matrix and the corresponding Al–TiC composites using a reciprocating ball-on-flat sliding wear mechanism at wear loads of 5 and 25 N.

Figure 11a shows the volumetric wear rates of the hot-rolled Al matrix and its composites reinforced with 3, 6, and 12 vol% TiC under wear loads varying from 5 to 25 N with increments of 5 N. Figure 11b presents an enlarged image of the selected area in Fig. 11a. The wear track widths of the hot-rolled Al matrix and their corresponding composites at the applied wear loads of 5 and 25 N are illustrated in Fig. 12. It can be observed that the volumetric wear rates and wear track widths of the wear-tested samples increased with increasing applied wear loads, as shown in Figs. 11 and 12, respectively. The hot-rolled Al–TiC composite strips exhibited noticeably lower volumetric wear rates than the Al matrix at all applied wear loads. Moreover, the volumetric wear rates of the Al matrix increased rapidly with increasing wear load compared to those of the Al–TiC composites (Fig. 11a). In addition, when the concentration of TiC ceramic particles was increased from 3 to 12 vol%, the wear resistance of the hot-rolled composites improved even further. Previous studies^9,39,51,52^ have also revealed a comparable trend in the wear rate behavior. The volumetric wear rate and depth of the wear track are typically described as the inverse of wear resistance. Consequently, the wear resistance of hot-rolled Al–TiC composites, to a great extent, improves as the addition of TiC particles increases, which might be due to the following: (i) significant improvement in the hardness of the hot-rolled composites compared to the matrix (Fig. 10); (ii) the presence of TiC particles in the composite shares the effective contact area between the Al matrix and the counter ball during the wear test, leading to improved wear resistance; and (iii) strengthening of the matrix, improving load-bearing capacity, and preventing thermally caused deformation with increased TiC concentration in the matrix. As mentioned previously, the wear regimes in the current study were applied at wear loads ranging from 5 to 25 N. The hot-rolled composites displayed noteworthy wear resistance at all applied wear loads compared to the hot-rolled matrix. For the hot-rolled Al-12 vol% TiC composite sample with the highest wear resistance (lowest volumetric wear rate), the enhancement in wear resistance is around 95% at the low load (5 N) in that applied wear regime and around 89% under the high applied wear load of 25 N compared to the hot-rolled Al-matrix, as shown in Fig. 11. In addition, the wear track widths of the Al matrix were 2.743 ± 0.432 and 4.421 ± 0.581 mm for the tested wear samples at loads of 5 and 25 N, respectively. After adding TiC particles to the matrix during the hot-rolling process, the widths of the wear tracks were reduced to 76% and 62%, respectively, compared to those of the Al matrix, as shown in Fig. 12.

*Wear mechanisms*

**Fig. 13:**
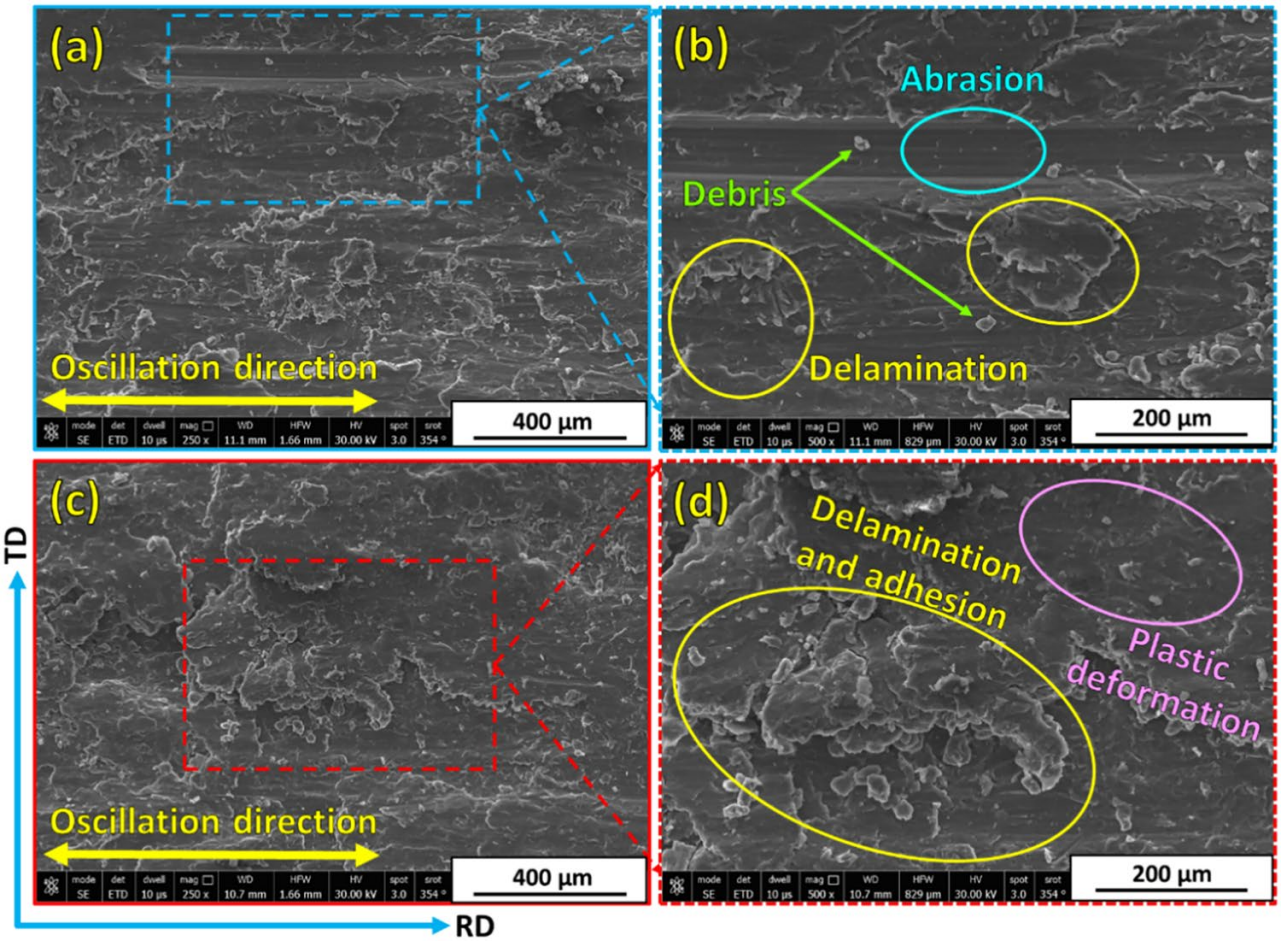
FE- SEM images (low and high magnifications) of worn surfaces subjected to loads of (**a**, **b**) 5 N and (**c**, **d**) 25 N for hot-rolled Al matrix.

**Fig. 14:**
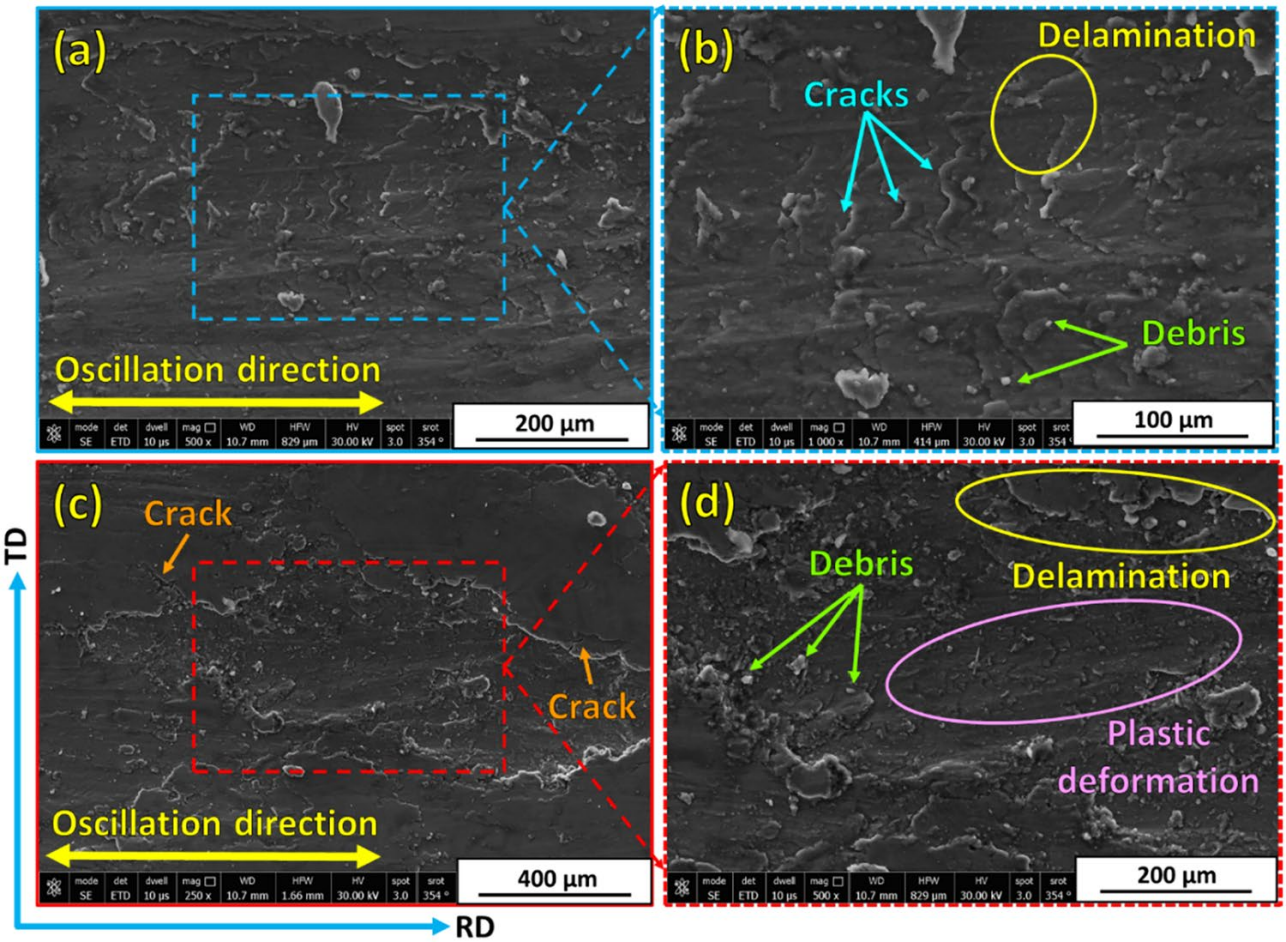
Worn surfaces at (**a**, **c**) low and (**b**, **d**) high magnifications of hot-rolled Al-3 vol% TiC composite subjected to wear loads of (**a**, **b**) 5 N and (**c**, **d**) 25 N.

**Fig. 15:**
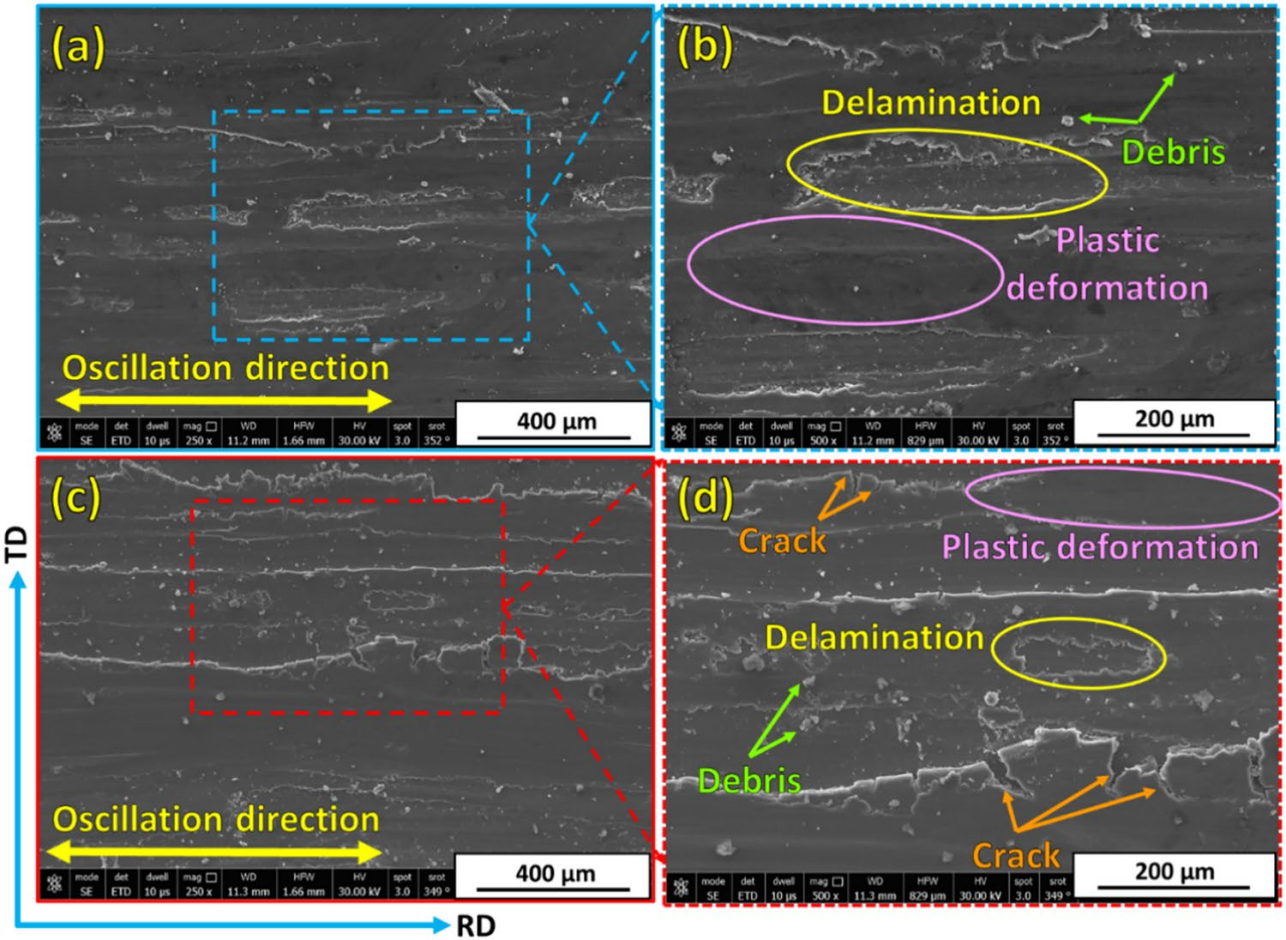
(**a**, **c**) Low and (**b**, **d**) high magnification images of the worn surfaces of wear-tested hot-rolled Al-6 vol% TiC composite under wear loads of (**a**, **b**) 5 N and (**c**, **d**) 25 N.

**Fig. 16:**
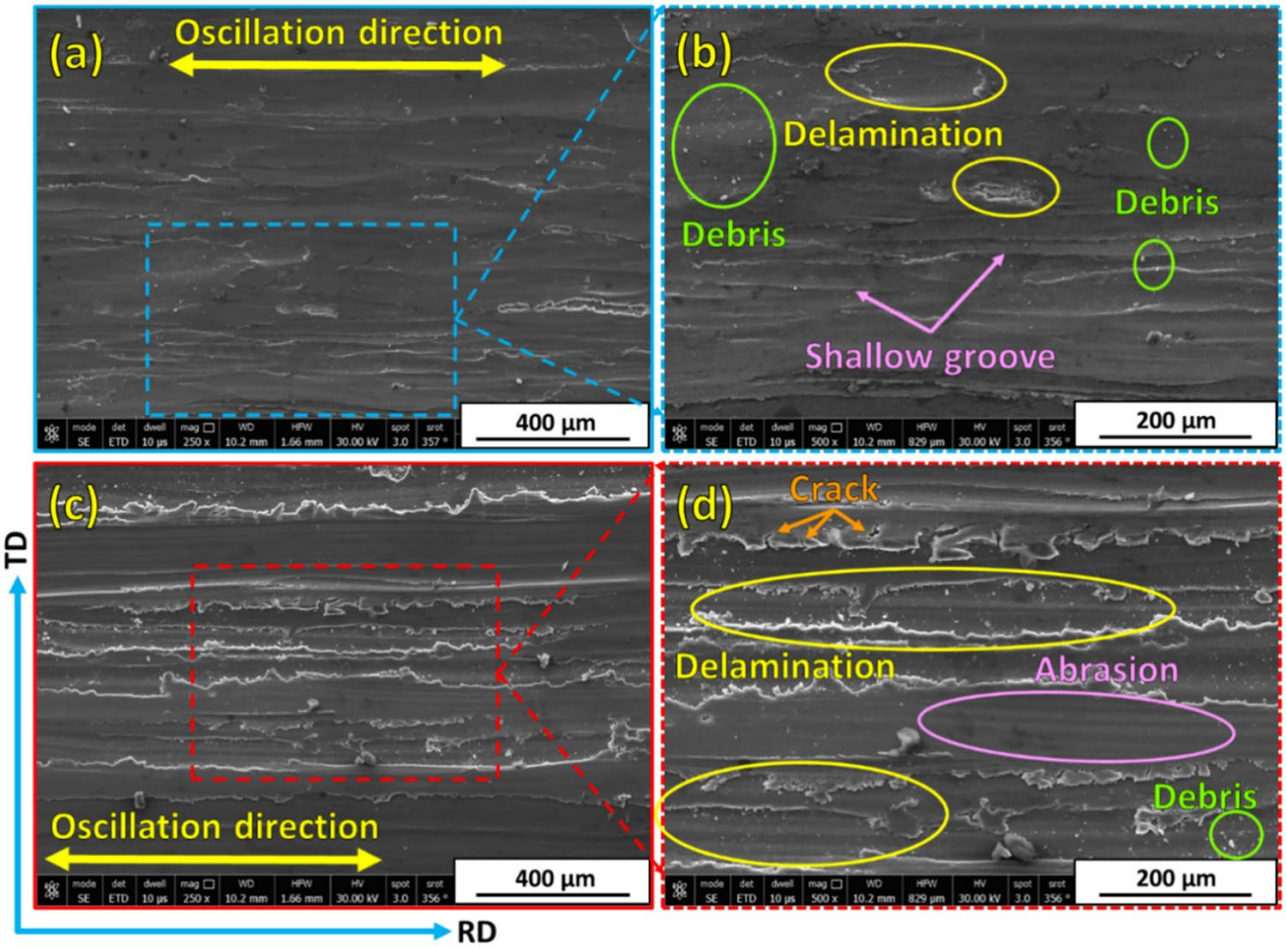
FE- SEM images at (**a**, **c**) low and (**b**, **d**) high magnifications of worn surfaces for the wear-tested Al-12 vol% composite strip at wear loads of (**a**, **b**) 5 N and (**c**, **d**) 25 N.

Examining and analyzing wear debris and studying wear surfaces are key techniques for determining the type of wear mechanism. To explore the wear mechanisms of hot-rolled Al matrix and its composites reinforced with 3, 6, and 12 vol% TiC, the worn surfaces of each sample after the wear tests under wear loads of 5N and 25N are characterized by FE-SEM, given in Figs. 13, 14, 15 and 16. In an early investigation of delamination theory^53^, it was found that the delamination process involves four distinct steps: (1) cyclic plastic deformation at the surface, (2) crack or void formation, (3) crack propagation, and (4) debris formation and removal. The present study found that increasing the TiC volume fraction hindered complete delamination wear. An increase in TiC content from 0 to 12 vol% retards the delamination wear to a great extent and subsequently decreases its wear rate, which is confirmed in Fig. 11. Two wear mechanisms can be observed in the hot-rolled Al matrix: abrasion, which is observed through the presence of groove marks parallel to the oscillation direction, and delamination, which is the dominant wear mechanism. Under the 5 N wear load, by increasing the volume percentage of reinforced TiC in the composite to 3 vol% and when under the 5 N wear load, cracks perpendicular to the sliding direction were observed, Fig. 14a,b. It has been reported in^4,54^ that cracking during the wear test of Al-matrix composites could be due to extensive plastic deformation of the Al matrix and/or particle–matrix interface debonding, which might lead to delamination and material removal. The addition of 3 vol% of TiC was capable of retarding delamination from taking place to its final step. However, the addition of 6 and 12 vol% %, under the same relatively low wear load, caused an increase in plastic deformation areas over a complete delamination process. For example, using 6 vol% TiC (Fig. 15a,b), the final delamination feature was nearly diminished compared with that of the hot-rolled Al-matrix (Fig. 13a,b). During the wear test, plastic deformation with shallow grooves was observed in Al-based composites reinforced with 12 vol% TiC that could be attributed to two main reasons: First, compared to the hot-rolled Al-matrix strip, the hardness of the composites with hard TiC particles is higher. This increase in hardness enhances the wear resistance by increasing the plastic deformation resistance during the sliding wear process, thereby reducing the wear rate^44,55^. Therefore, including hard TiC particles in the hot-rolled composite strip improved its hardness (Fig. 10) and wear resistance (Fig. 11). This implies that when the hot-rolled composite was subjected to a wear load, the TiC particles resisted deformation, whereas the surrounding Al matrix deformed. This can lead to the formation of shallow grooves on the worn surface of the composite. Second, TiC ceramic particles were distributed throughout the Al matrix. This implies that the TiC particles constrained the plastic deformation of the Al matrix. This can lead to the formation of shallow grooves instead of severe deformation. At a high wear load (25 N), substantial plastic deformation of the wear surface was observed for all materials particularly for materials with higher TiC contents. Similar to what was found when under a 5N wear load, the main wear mechanism is delamination wear. Severe delamination was detected in the unreinforced hot-rolled Al-matrix strip (Fig. 13c,d), which may indicate adhesive wear, as stated in^44^. In addition, several areas of plastic deformation were observed. Increasing the TiC content to 3 vol%, 6 vol%, and 12 vol%, the wear track and surface tribology become smoother, and plastic deformation areas were increased, as shown in Figs. 14c,d, 15c,d, and 16c,d, respectively. At higher TiC contents, cracks almost perpendicular to the sliding direction on the delaminated surface edges were observed, as shown in Figs. 15d and 16d. These cracks became significant in the case of the 12 vol% TiC composite, as shown in Fig. 16c,d. The presence of such cracks could be due to the severe plastic deformation encountered under the 25 N wear load and/or cracking of the Al-based oxide layer formed at the surface because of frictional heat during dry sliding wear^56^. Although oxide layers in Al-based alloys are believed to delay the transition between mild and severe wear, cracking in the surface oxide layer, on the other hand, enhances the delamination process and might lead to the establishment of a severe wear mechanism^56^. Although mechanical alloying is known to reduce the effect of particle/intermetallic agglomeration in composites, TiC agglomerates were detected after the hot-rolling process^44,45^, as shown in Fig. 7. Although segregation is not severe, it might induce easy spalling of the titanium carbide particles, which can function as abrasives and thus enhance abrasive wear, as shown in Fig. 16d ^44^.

**Fig. 17:**
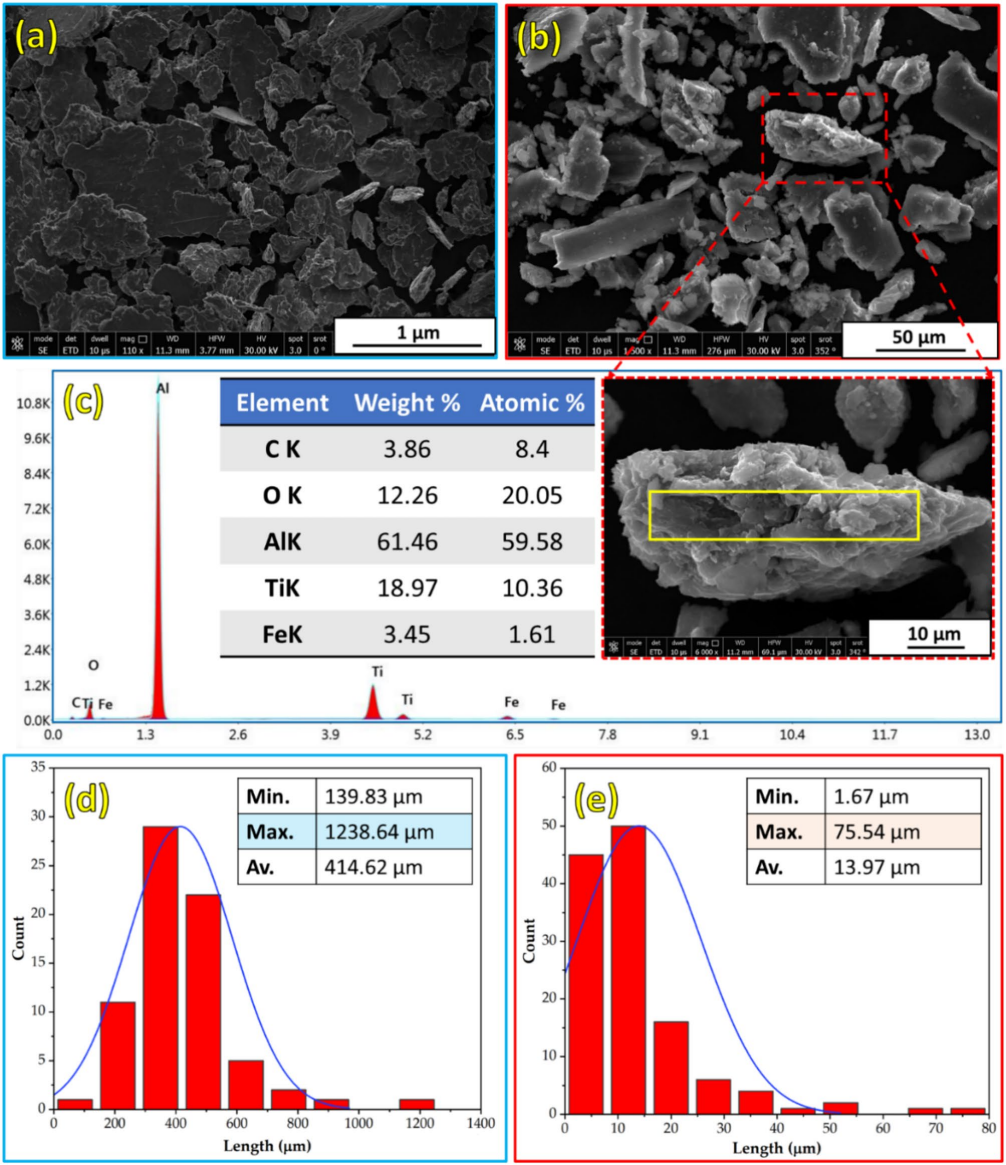
FE-SEM images of debris collected at a wear load of 25 N for the hot-rolled (**a**) Al matrix and (**b**) Al-12 vol% TiC composite, (**c**) EDS analysis of the collected debris, and size analysis of the collected debris for the hot-rolled (**d**) Al-matrix and (**e**) Al-12 vol% TiC composite.

The wear debris generated from the Al matrix and Al-12 vol% TiC composite strips were collected and analyzed using the FE-SEM images with the EDS and size analysis to support the detected wear performance and their active wear mechanisms in each wear-tested hot rolled strip. Figure 17 shows the FE-SEM, EDS, and size of the collected debris for the hot-rolled Al matrix and the Al-12 vol% TiC composite after the dry wear test at a load of 25 N. Figure 17a illustrates the presence of many sizable pieces of debris with distinct large and irregular flake shapes, which can be easily discerned without magnification. This observation provides evidence of adhesion and delamination wear. Large flake-shaped wear debris can be established from the intensive adhesion and delamination wear of worn surfaces^44^. In addition, shallow flow marks on some large-sized debris were found in the hot-rolled Al matrix material but not in the composite, indicating substantial plastic deformation during the wear test^57,58^. Upon comparing Fig. 17a,b, it can be observed that the size of the delaminated wear debris generated by the unreinforced hot-rolled strip ranges from 139.83 to 1238.64 µm with an average debris size of 414.62 µm (Fig. 17d). However, the size of the wear debris delaminated by the Al-12 vol% TiC hot-rolled composite strip ranges from 1.67 to 75.54 µm with an average debris size of 13.97 µm (Fig. 17e). The average size of the debris collected from the Al-12 vol% TiC hot-rolled composite strip is approximately 18% of the debris from the hot-rolled Al-matrix strip, as shown in Fig. 17d,e. This observation suggests that the TiC ceramic particles substantially affected the size and morphology of the generated wear debris during the sliding wear test, causing a transformation from larger to smaller debris. Furthermore, the decrease in the debris size was notably more pronounced when hot-rolled composite strips with a high concentration of reinforcements were considered. The EDS analysis revealed the nature of the debris collected from the 12% TiC composite. Moreover, the presence of peaks in titanium (Ti) and carbon (C) was detected by EDS, as depicted in Fig. 17c. This observation may be associated with TiC particle pullout or fracture occurring during the wear test. Furthermore, a significant amount of oxygen was present, which may indicate an oxidation-wear mechanism. In addition, the observation of an iron (Fe) peak (Fig. 17c) is evidence of wear that occurred on the counter-ball surface and was transferred to the developed debris during the wear test of the hot-rolled composite strips.

**Fig. 18:**
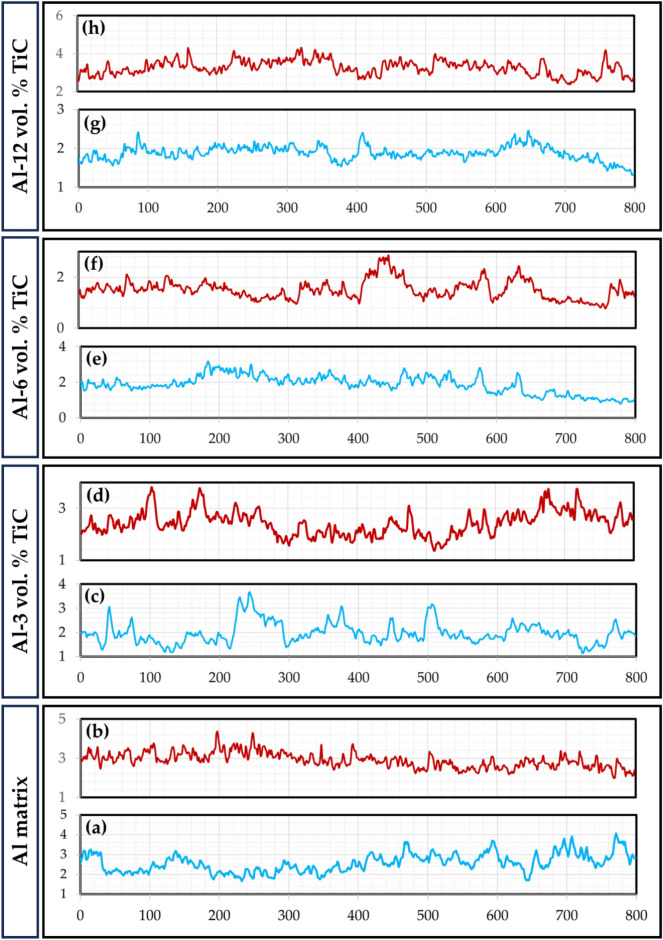
Roughness profiles of the hot-rolled (**a** and **b**) Al matrix and their (**c**–**h**) Al–TiC composite strips using reciprocating ball-on-flat sliding wear mechanism at wear loads of 5 N (blue profiles) and 25 N (red profiles).

**Fig. 19:**
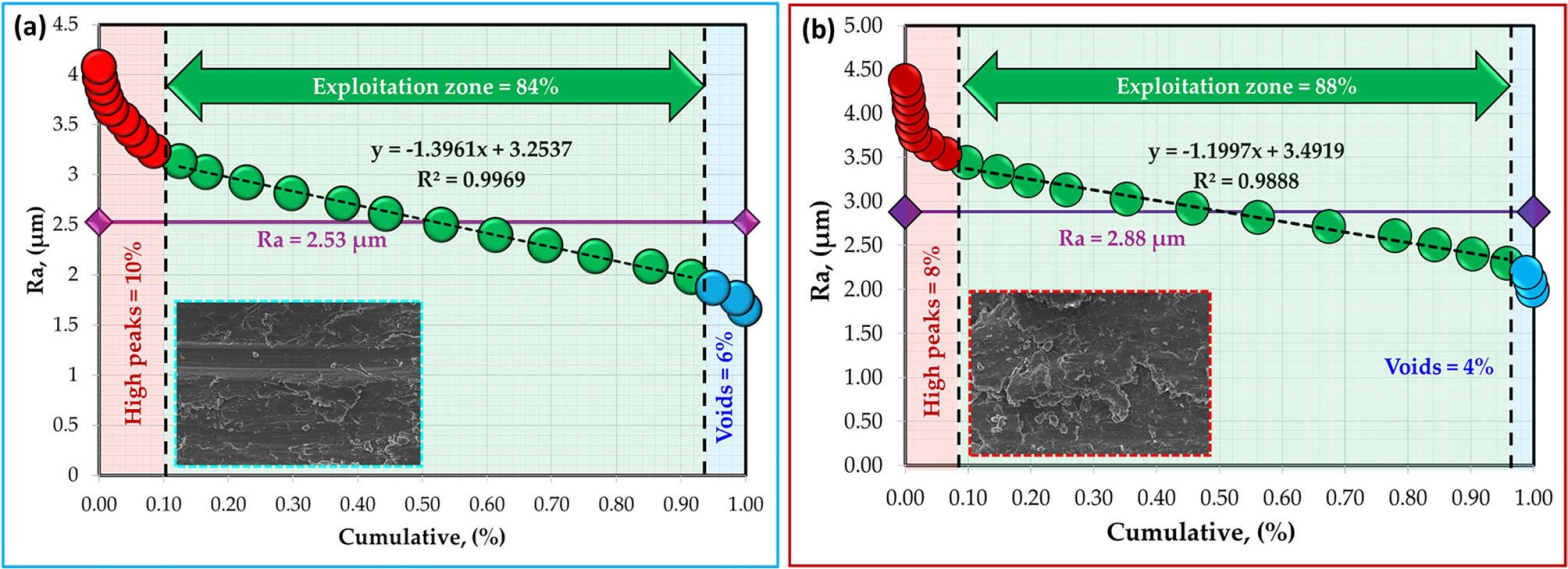
Abbott-Firestone curves of hot-rolled Al-matrix strip subjected to wear loads of (**a**) 5 N and (**b**) 25 N.

**Fig. 20:**
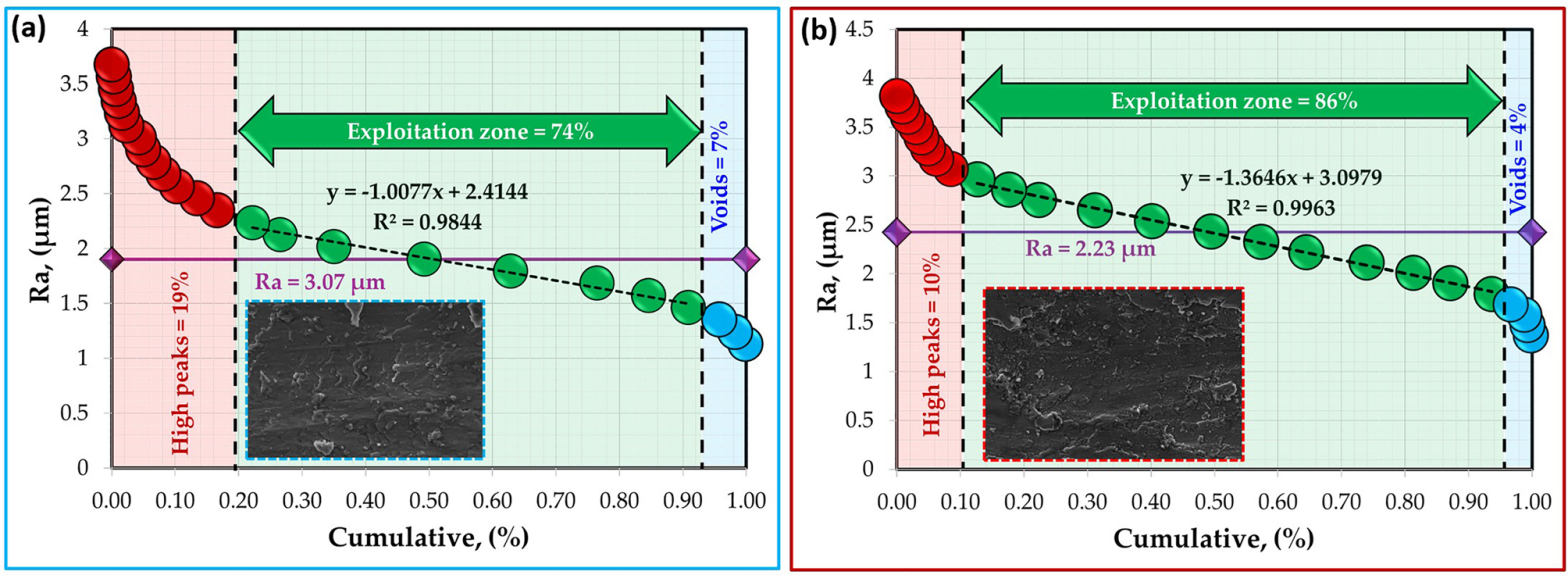
Abbott-Firestone curves of hot-rolled Al-3 vol% TiC composite strip subjected to wear loads of (**a**) 5 N and (**b**) 25 N.

**Fig. 21:**
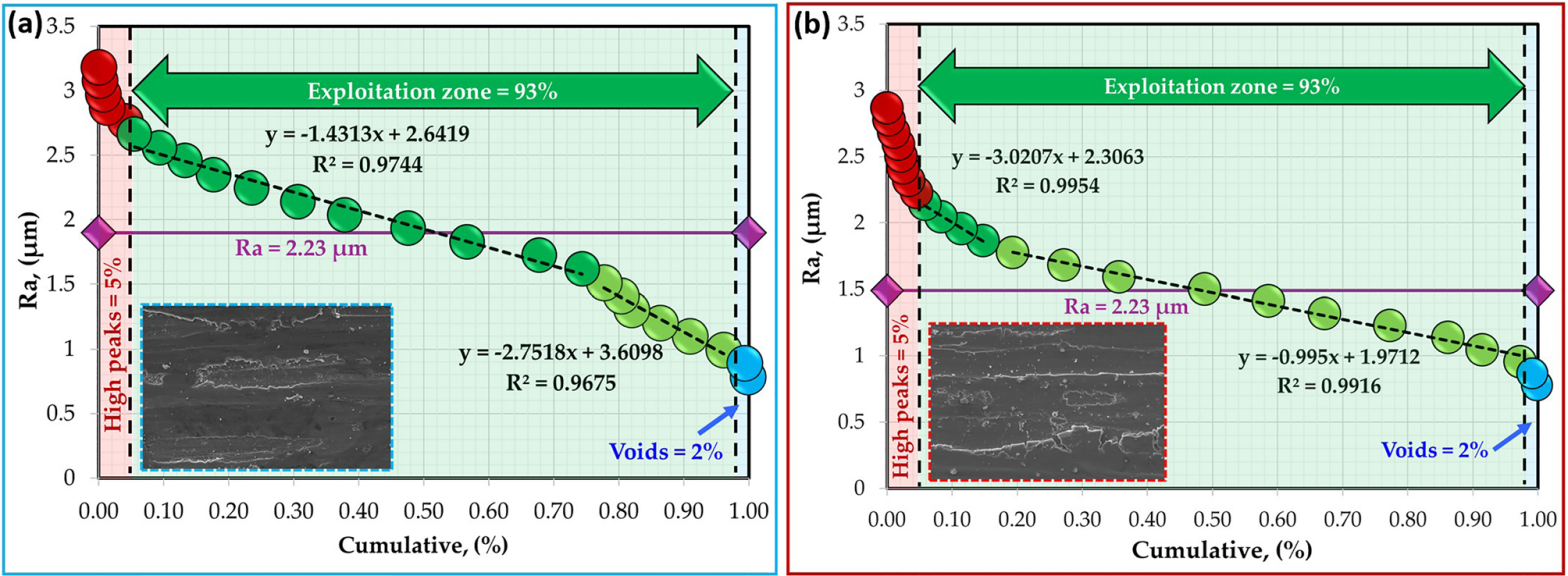
Abbott-Firestone curves of hot-rolled Al-6 vol% TiC composite strip subjected to wear loads of (**a**) 5 N and (**b**) 25 N.

**Fig. 22:**
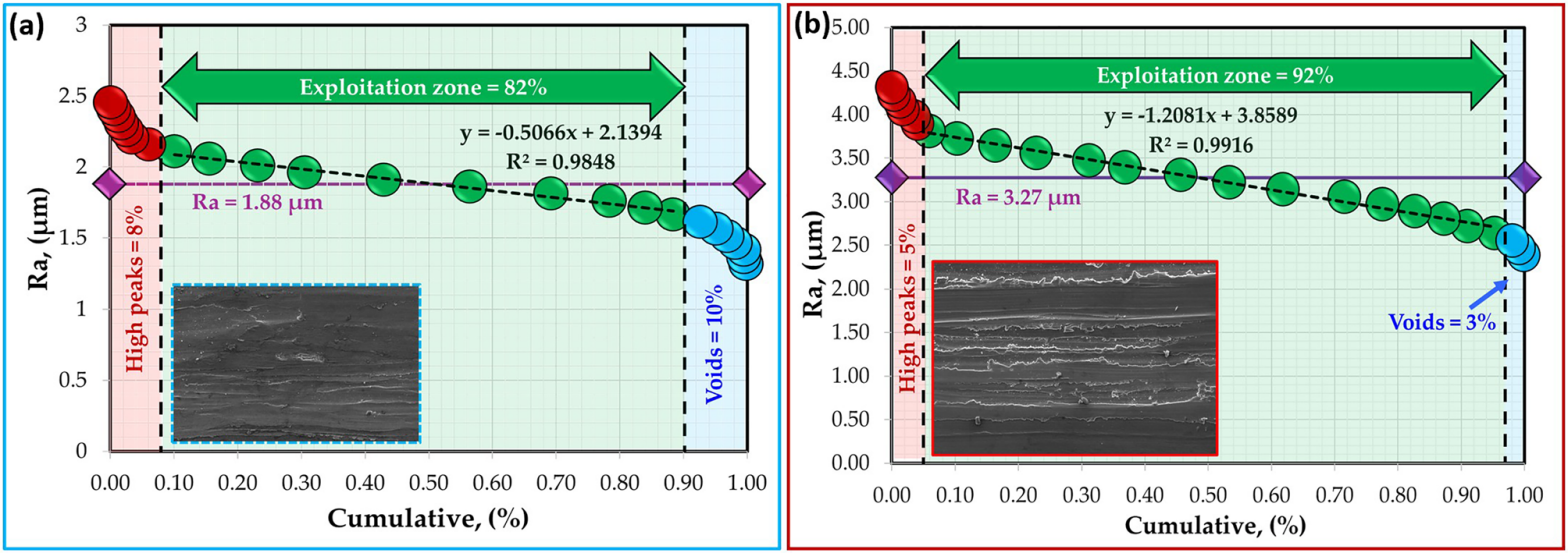
Abbott-Firestone curves of hot-rolled Al-12 vol% TiC composite strip subjected to wear loads of (**a**) 5 N and (**b**) 25 N.

The surface roughness profiles of the worn surfaces of the hot-rolled Al strips and their Al–TiC composite strips are shown in Fig. 18. The analyses of worn surfaces are conducted through Abbott-Firestone curves (AFCs). Such curves play a vital role in wear surface analysis, especially in characterizing the functional parts of the wear and in predicting its effect on the performance of the tested samples. Figures 19, 20, 21 and 22 describe the AFCs of the wear-tested samples of the hot-rolled Al matrix and their corresponding Al–TiC composites. The AFCs allow for the identification of three distinct wear zones: **(1)** High peaks, which represent roughness that is susceptible to initial wear but may not significantly affect the overall performance of the materials. **(2)** Central exploitation zone: This critical area describes the functional contact area between surfaces and is crucial for lubrication, load-bearing capacity, and friction. **(3)** Voids: These represent areas that might not directly contribute to the wear resistance functionality but can influence factors such as lubricant retention or debris. The roughness profiles of all wear-tested samples ranged from 1 to 5 µm, as shown in Fig. 18. At a lower wear load of 5 N, the average roughness (Ra) of the hot-rolled Al matrix and the corresponding Al–TiC composite strips increased with the addition of 3 vol% TiC from 2.53 to 3.07 um. Subsequently, Ra gradually decreased to 1.88 um with the further addition of TiC up to 12 vol%. In addition, the Ra of the hot-rolled strips decreased from 2.88 to 2.23 um with the addition of TiC particles up to 6 vol%, and then increased to 3.27 um with a TiC content of 12 vol%, as illustrated in Fig. 18. From the AFCs (Figs. 19, 20, 21 and 22), it can be noted that a lower high-peak area is observed in the hot-rolled Al-6 vol% TiC composite strip. A high-peak zone represents the highest asperities or peaks on the worn surface. These peaks are typically the first points of contact that experience initial wear and often experience higher wear rates than the exploitation zone. For relatively low peaks, the contact area typically shifts towards the exploitation zone. The results of the AFCs revealed that all hot-rolled Al matrices and the corresponding composite strips had a wide exploitation zone ranging from 74 to 93%. Therefore, while the high-peak zone plays an initial role in wear by experiencing early wear, its impact on the overall wear rate and load distribution is limited compared with that of the exploitation zone. The highest central exploitation zone was detected in the hot-rolled strips reinforced with 6 wt% TiC. A large exploitation zone was found to distribute the load evenly. The stress concentration at any single point was reduced by distributing the load over a larger area, resulting in a lower overall wear rate. In contrast, a smaller exploitation zone concentrates the load over a smaller area, which leads to localized high wear, potentially causing deeper wear grooves or increased wear rates in specific regions. However, the overall wear rate across the surface was expected to be higher than that across a larger exploitation zone. In addition, the void area in the AFC represents the deepest depression or valley on the surface. Although they do not directly contribute to wear compared to the exploitation zone or high peaks, they can still play a significant role in the overall performance of a tribological system. Deep valleys can trap debris, leading to abrasive wear if they are in contact with the exploitation zone. The smallest void area was observed for the hot-rolled Al-6 vol% TiC composite strip.

### Limitations, broader contributions, and future directions

Despite offering significant information regarding the fabrication and characteristics of Al–TiC composite strips, this study has several limitations. The present study focuses on TiC concentrations up to 12 vol% but exploring higher concentrations could provide additional insights into the upper limits of property enhancement and low concentrations with small increments. Further, the study employed a fixed set of hot-rolling parameters, while investigating the effects of varying rolling temperatures, speeds, and reduction ratios could further optimize the process. Additionally, wear tests were conducted over relatively short durations, and the long-term wear behavior and fatigue performance remain to be studied. Despite these limitations, the present study contributes to the broader field of composite materials in several ways. For example, the current study demonstrates the effectiveness of combining mechanical alloying and hot rolling for producing high-quality Al–TiC composites, which could be applicable to other metal matrix composites. Additionally, the current findings provide valuable insights into how TiC reinforcement affects the microstructure and the resulting mechanical and tribological properties of Al–TiC composite strips. The improved properties of Al–TiC composite strips make them promising candidates for various automotive components. In light of the study’s results, the following recommendations for future research are suggested. Additional research should concentrate on optimizing the mechanical alloying and hot-rolling parameters for property enhancements. Furthermore, examining the effects of TiC concentration could help in determining the optimal reinforcement content for specific applications. In addition, combining TiC with other reinforcing substances (e.g., carbon nanotubes and graphene) could potentially lead to synergistic property improvements. Equally important, investigating the tensile properties and corrosion resistance of these composites is crucial for their application in the automotive and other industries. Finally, studying the thermal conductivity and coefficient of thermal expansion of these composites would be valuable for their potential use in thermal management applications. These future research directions could further advance our understanding and application of Al–TiC composites, potentially leading to new materials with enhanced performance for various engineering applications.

## Conclusions

This study aimed to assess the impact of a multi-step production technique and the addition of TiC on several aspects of hot-rolled Al–TiC composite strips, including the microstructure, densification, hardness, and tribological performance. Based on the experimental results obtained from various investigations conducted on hot-rolled Al-matrix strips and their composite strips, the following conclusions can be drawn:

1. The applied production method was successful in fabricating Al–TiC composite strips reinforced with different concentrations ranging from 0 to 12 vol% with a near-uniform distribution of TiC particles in the hot-rolled strips, which was confirmed by the XRD, FE-SEM, mapping, and EDS analyses.
2. The densities of the hot-rolled composite strips closely approached full densification, with values ranging from 99.28 to 100.00% for the hot-rolled Al-12 vol% TiC and Al-matrix strips, respectively. The porosity of the hot-rolled composite strips increased to 0.72% with the addition of TiC at a concentration of 12 vol%, representing very low levels of porosity.
3. The hardness of the hot-rolled composite strips was higher than that of the Al matrix strip, and the greatest improvement was observed for the hot-rolled AL-12 vol% TiC strip with an enhancement of ~ 268,6% compared to the Al-matrix strip.
4. The addition of TiC ceramic particles significantly enhanced the wear resistance of hot-rolled composites. The hot-rolled Al-12 vol% strip demonstrated superior wear performance under the applied load regime, around 95% and 89% under the low (5 N) and high (25 N) applied wear loads, respectively, compared to the hot-rolled Al-matrix.
5. The dominant wear mechanism of the Al matrix strip was abrasion. However, as the applied load increased, the wear mechanism transitioned to delamination and adhesion. The addition of TiC particles hindered the formation of complete delamination wear in the hot-rolled composite strips.
6. The evaluation of the worn surface indicates that the exploitation zone plays a crucial role in preventing material failure. The results showed that a hot-rolled Al–TiC composite strip reinforced with 6 vol% TiC particles exhibited a higher exploitation zone at both low and high wear load conditions.

## Data Availability

The datasets used and/or analyzed during the current study available from the corresponding author on reasonable request.

## References

[1] Singh, R., Sharma, A. K., Sharma, A. K. & Shukla, M. N. Fabrication of Al–TiC composites by three-step powder metallurgy: Microstructure and mechanical properties. *Int. J. Veh. Struct. Syst.* **14**, 634–639 (2022).

[2] Habba, M. I. A., Barakat, W. S. & Hamid, F. S. Experimental investigation and numerical modeling of mechanically alloyed Al–TiC composites. *J. Compos. Mater.* **57**(22), 3529–3556 (2023). 10.1177/00219983231189551

[3] Ramasamy, M., Daniel, A. A. & Nithya, M. A review on aluminium (Al7050) metal matrix composite characteristics reinforced with titanium. *Mater. Today Proc.* **43**, 1720–1723. 10.1016/j.matpr.2020.10.257 (2020). 10.1016/j.matpr.2020.10.257

[4] Seshan, S., Guruprasad, A., Prabha, M. & Sudhakar, A. Fibre-reinforced metal matrix composites - A review. *J. Indian Inst. Sci.* **76**, 1–14 (1996).

[5] Barakat, W. S., Younis, M. K., Sadoun, A. M., Fathy, A. & Habba, M. I. Optimization of the accumulative roll bonding process parameters and SiC content for optimum enhancement in mechanical properties of Al-Ni-SiC composites. *Alexandria Eng. J.* **76**, 131–151 (2023). 10.1016/j.aej.2023.06.027

[6] Chakrapani, P. & Suryakumari, T. S. A. Mechanical properties of aluminium metal matrix composites-A review. *Mater. Today Proc.*10.1016/j.matpr.2020.09.247 (2020). 10.1016/j.matpr.2020.09.247

[7] Habba, M. I. A., Younis, M. K., Sadoun, A. M., Fathy, A. & Barakat, W. S. On the microstructural and mechanical responses of dual-matrix Al-Ni/SiC composites manufactured using accumulative roll bonding. *Alexandria Eng. J.* **78**, 1–14 (2023). 10.1016/j.aej.2023.07.030

[8] Rahiman, A. H. S. *et al.* Dry sliding wear analysis of Al5083/CNT/Ni/MoB hybrid composite using DOE Taguchi method. *Wear* **460–461**, 203471 (2020). 10.1016/j.wear.2020.203471

[9] Tang, F. *et al.* Dry sliding friction and wear properties of B4C particulate-reinforced Al-5083 matrix composites. *Wear* **264**, 555–561 (2008). 10.1016/j.wear.2007.04.006

[10] Siddhalingeshwar, I. G., Deepthi, D., Chakraborty, M. & Mitra, R. Sliding wear behavior of in situ Al-4.5Cu-5TiB2 composite processed by single and multiple roll passes in mushy state. *Wear* **271**, 748–759 (2011). 10.1016/j.wear.2011.03.017

[11] Barakat, W. S., Habba, M. I. A., Ibrahim, A., Fathy, A. & Elkady, O. A. The effect of Cu coated Al2O3 particle content and densification methods on the microstructure and mechanical properties of Al matrix composites. *J. Mater. Res. Technol.* **24**, 6908–6922 (2023). 10.1016/j.jmrt.2023.05.010

[12] Sattari, S., Jahani, M. & Atrian, A. Effect of volume fraction of reinforcement and milling time on physical and mechanical properties of Al7075–SiC composites fabricated by powder metallurgy method. *Powder Metall. Met. Ceram.* **56**, 283–292 (2017). 10.1007/s11106-017-9896-2

[13] Wang, W., Heydari Vini, M. & Daneshmand, S. Mechanical and wear properties of Al/TiC composites fabricated via combined compo-casting and APB process. *Crystals* **12**, 1440 (2022). 10.3390/cryst12101440

[14] Sathiyaraj, S., Senthilkumar, A., Muhammed Ameen, P., Sundar, R. & Saseendran, V. Experimental investigations on mechanical properties of Al-B4C metal matrix composites. *Mater. Today Proc.*10.1016/j.matpr.2020.11.017 (2021). 10.1016/j.matpr.2020.11.017

[15] Boppana, S. B., Dayanand, S., Anil Kumar, M. R., Kumar, V. & Aravinda, T. Synthesis and characterization of nano graphene and ZrO2 reinforced Al 6061 metal matrix composites. *J. Mater. Res. Technol.* **9**, 7354–7362 (2020). 10.1016/j.jmrt.2020.05.013

[16] Jerome, S., Ravisankar, B., Kumar Mahato, P. & Natarajan, S. Synthesis and evaluation of mechanical and high temperature tribological properties of in-situ AlTiC composites. *Tribol. Int.* **43**, 2029–2036 (2010). 10.1016/j.triboint.2010.05.007

[17] Tyagi, R. Synthesis and tribological characterization of in situ cast Al-TiC composites. *Wear* **259**, 569–576 (2005). 10.1016/j.wear.2005.01.051

[18] Nyanor, P., El-Kady, O., Yehia, H. M., Hamada, A. S. & Hassan, M. A. Effect of bimodal-sized hybrid TiC–CNT reinforcement on the mechanical properties and coefficient of thermal expansion of aluminium matrix composites. *Met. Mater. Int.* **27**, 753–766 (2021). 10.1007/s12540-020-00802-w

[19] Whalen, S. *et al.* High ductility aluminum alloy made from powder by friction extrusion. *Materialia* **6**, 100260 (2019). 10.1016/j.mtla.2019.100260

[20] Yu, W. *et al.* Tribological and texture analysis in Twin-roll casting 2060 Al-Li alloy. *Mater. Des.* **223**, 111253 (2022). 10.1016/j.matdes.2022.111253

[21] Li, J. *et al.* Preparation of Mg2Si/Al–Cu composite under a novel continuous squeeze casting-extrusion process assisted with ultrasonic treatment. *Mater. Sci. Eng. A* **862**, 144469 (2023). 10.1016/j.msea.2022.144469

[22] Farhadipour, P., Sedighi, M. & Heydari Vini, M. Influence of temperature of accumulative roll bonding on the mechanical properties of AA5083–1% Al2O3 composite. *Powder Metall. Met. Ceram.* **56**, 496–503 (2018). 10.1007/s11106-018-9921-0

[23] Peat, T., Galloway, A., Toumpis, A., McNutt, P. & Iqbal, N. The erosion performance of particle reinforced metal matrix composite coatings produced by co-deposition cold gas dynamic spraying. *Appl. Surf. Sci.* **396**, 1623–1634 (2017). 10.1016/j.apsusc.2016.10.155

[24] Wagih, A. & Fathy, A. Improving compressibility and thermal properties of Al–Al2O3 nanocomposites using Mg particles. *J. Mater. Sci.* **53**, 11393–11402 (2018). 10.1007/s10853-018-2422-1

[25] Zebarjad, S. M. & Sajjadi, S. A. Microstructure evaluation of Al-Al2O3 composite produced by mechanical alloying method. *Mater. Des.* **27**, 684–688 (2006). 10.1016/j.matdes.2004.12.011

[26] Barakat, W., Elkady, O., Abuoqail, A., Yehya, H. & El-Nikhailay, A. Effect of Al2O3 coated Cu nanoparticles on properties OF Al/Al2O3 composites. *J. Pet. Min. Eng.* **22**(1), 1–8 (2020).

[27] Bhattacharyya, J. J. & Mitra, R. Effect of hot rolling temperature and thermal cycling on creep and damage behavior of powder metallurgy processed Al-SiC particulate composite. *Mater. Sci. Eng. A* **557**, 92–105 (2012). 10.1016/j.msea.2012.06.073

[28] Fei, W. D., Li, W. Z. & Yao, C. K. Hot rolling behaviors of whisker reinforced aluminum composites. *J. Mater. Sci.* **37**, 211–215 (2002). 10.1023/A:1013199320555

[29] Nayak, K. C. & Date, P. P. Physical simulation of hot rolling of powder metallurgy-based Al/SiC composite by plane strain multi stage compression. *Mater. Charact.* **173**, 110954 (2021). 10.1016/j.matchar.2021.110954

[30] Li, X. P., Liu, C. Y., Ma, M. Z. & Liu, R. P. Microstructures and mechanical properties of AA6061-SiC composites prepared through spark plasma sintering and hot rolling. *Mater. Sci. Eng. A* **650**, 139–144 (2016). 10.1016/j.msea.2015.10.015

[31] Yuan, C. *et al.* Fabrication and mechanical properties of CNT/Al composites via shift-speed ball milling and hot-rolling. *J. Mater. Res.* **34**, 2609–2619 (2019). 10.1557/jmr.2019.219

[32] Zabihi, M., Toroghinejad, M. R. & Shafyei, A. Application of powder metallurgy and hot rolling processes for manufacturing aluminum/alumina composite strips. *Mater. Sci. Eng. A* **560**, 567–574 (2013). 10.1016/j.msea.2012.09.103

[33] Lekatou, A. G., Sfikas, A. K., Sioulas, D. & Kanderakis, A. Sliding wear performance of Al–Co alloys fabricated by vacuum arc melting and correlation with their microstructure. *Mater. Chem. Phys.* **276**, 125411 (2022). 10.1016/j.matchemphys.2021.125411

[34] Hashimoto, S., Yamaguchi, A. & Koshino, M. Fabrication and characterization of TiC/Al composites. *Mater. Sci. Eng. A* **265**, 71–76 (1999). 10.1016/S0921-5093(99)00005-2

[35] Samer, N. *et al.* Microstructure and mechanical properties of an Al-TiC metal matrix composite obtained by reactive synthesis. *Compos. Part A Appl. Sci. Manuf.* **72**, 50–57 (2015). 10.1016/j.compositesa.2015.02.001

[36] Aborkin, A., Babin, D. & Bokaryov, D. Control of Al4C3 phase formation in aluminum matrix composites reinforced with carbon nanostructures. *E3S Web Conf.* **431**, 1–9 (2023). 10.1051/e3sconf/202343106012

[37] Khdair, A. I. & Fathy, A. Enhanced strength and ductility of Al-SiC nanocomposites synthesized by accumulative roll bonding. *J. Mater. Res. Technol.* **9**, 478–489 (2020). 10.1016/j.jmrt.2019.10.077

[38] Sadeghi, B. *et al.* Hot rolling of MWCNTs reinforced Al matrix composites produced via spark plasma sintering. *Adv. Compos. Hybrid Mater.* **2**, 549–570 (2019). 10.1007/s42114-019-00095-7

[39] Banijamali, S. M., Najafi, S., Sheikhani, A. & Palizdar, Y. Dry tribological behavior of hot-rolled WE43 magnesium matrix composites reinforced by B4C particles. *Wear* **508–509**, 204487 (2022). 10.1016/j.wear.2022.204487

[40] Wang, P. *et al.* Fabrication of Ti/Al/Mg laminated composites by hot roll bonding and their microstructures and mechanical properties. *Chin. J. Aeronaut.* **34**, 192–201 (2021). 10.1016/j.cja.2020.08.044

[41] Azimi, A., Shokuhfar, A. & Nejadseyfi, O. Mechanically alloyed Al7075-TiC nanocomposite: Powder processing, consolidation and mechanical strength. *Mater. Des.* **66**, 137–141 (2015). 10.1016/j.matdes.2014.10.046

[42] Jeyasimman, D., Sivasankaran, S., Sivaprasad, K., Narayanasamy, R. & Kambali, R. S. An investigation of the synthesis, consolidation and mechanical behaviour of Al 6061 nanocomposites reinforced by TiC via mechanical alloying. *Mater. Des.* **57**, 394–404 (2014). 10.1016/j.matdes.2013.12.067

[43] Feijoo, I. *et al.* Estimation of crystallite size and lattice strain in nano-sized TiC particle-reinforced 6005A aluminium alloy from X-ray diffraction line broadening. *Powder Technol.* **343**, 19–28 (2019). 10.1016/j.powtec.2018.11.010

[44] Lin, F. *et al.* Fabrication of TiC-graphene dual-reinforced self-lubricating Al matrix hybrid nanocomposites with superior mechanical and tribological properties. *Tribol. Int.* **171**, 107535 (2022). 10.1016/j.triboint.2022.107535

[45] Nemati, N., Khosroshahi, R., Emamy, M. & Zolriasatein, A. Investigation of microstructure, hardness and wear properties of Al–4.5 wt% Cu–TiC nanocomposites produced by mechanical milling. *Mater. Des.* **32**, 3718–3729 (2011). 10.1016/j.matdes.2011.03.056

[46] Kumar, R. V., Keshavamurthy, R., Perugu, C. S., Koppad, P. G. & Alipour, M. Influence of hot rolling on microstructure and mechanical behaviour of Al6061-ZrB2 in-situ metal matrix composites. *Mater. Sci. Eng. A* **738**, 344–352 (2018). 10.1016/j.msea.2018.09.104

[47] Albert, T. *et al.* Preparation and characterization of aluminium-titanium carbide (Al-TiC) composite using powder metallurgy. *Mater. Today Proc.* **37**, 1558–1561 (2020). 10.1016/j.matpr.2020.07.155

[48] Sreeram, D., Pugazhenthi, R., Anbuchezhiyan, G., Saravanan, R. & Veeranjaneyulu, K. An investigation of the effects of hot rolling on the microstructure and mechanical behavior of nano-sized SiC particulates reinforced Al6063 alloy composites. *Mater. Today Proc.* **64**, 731–736 (2022). 10.1016/j.matpr.2022.05.197

[49] Guo, B. *et al.* Microstructures and mechanical properties of carbon nanotubes reinforced pure aluminum composites synthesized by spark plasma sintering and hot rolling. *Mater. Sci. Eng. A* **698**, 282–288 (2017). 10.1016/j.msea.2017.05.068

[50] Xu, S. C. *et al.* Evolution of texture during hot rolling of aluminum borate whisker-reinforced 6061 aluminum alloy composite. *Mater. Sci. Eng. A* **528**, 3243–3248 (2011). 10.1016/j.msea.2010.12.103

[51] Bastwros, M. M. H., Esawi, A. M. K. & Wifi, A. Friction and wear behavior of Al-CNT composites. *Wear* **307**, 164–173 (2013). 10.1016/j.wear.2013.08.021

[52] Rahmani, K., Majzoobi, G. H., Ebrahim-Zadeh, G. & Kashfi, M. Comprehensive study on quasi-static and dynamic mechanical properties and wear behavior of Mg—B4C composite compacted at several loading rates through powder metallurgy. *Trans. Nonferrous Met. Soc. China* **31**, 371–381 (2021). 10.1016/S1003-6326(21)65502-4

[53] Suh, N. P. The delamination theory of wear. *Wear* **25**, 111–124 (1973). 10.1016/0043-1648(73)90125-7

[54] Sharma, P., Paliwal, K., Garg, R. K., Sharma, S. & Khanduja, D. A study on wear behaviour of Al/6101/graphite composites. *J. Asian Ceram. Soc.* **5**, 42–48 (2017). 10.1016/j.jascer.2016.12.007

[55] Tyagi, R. Synthesis and tribological characterization of in situ cast Al–TiC composites. *Wear* **259**, 569–576 (2005). 10.1016/j.wear.2005.01.051

[56] Lekatou, A. *et al.* Aluminium reinforced by WC and TiC nanoparticles (ex-situ) and aluminide particles (in-situ): Microstructure, wear and corrosion behaviour. *Mater. Des.* **65**, 1121–1135 (2015). 10.1016/j.matdes.2014.08.040

[57] Sardar, S., Karmakar, S. K. & Das, D. High stress abrasive wear characteristics of Al 7075 alloy and 7075/Al2O3 composite. *Measurement* **127**, 42–62 (2018). 10.1016/j.measurement.2018.05.090

[58] Singh, J. & Chauhan, A. Overview of wear performance of aluminium matrix composites reinforced with ceramic materials under the influence of controllable variables. *Ceram. Int.* **42**, 56–81 (2016). 10.1016/j.ceramint.2015.08.150

[59] Alam, M. A. *et al.* Artificial neural network modeling to predict the effect of milling time and TiC content on the crystallite size and lattice strain of Al7075-TiC composites fabricated by powder metallurgy. *Crystals* **12**, 372 (2022). 10.3390/cryst12030372

[60] Cabeza, M. *et al.* Effect of high energy ball milling on the morphology, microstructure and properties of nano-sized TiC particle-reinforced 6005A aluminium alloy matrix composite. *Powder Technol.* **321**, 31–43 (2017). 10.1016/j.powtec.2017.07.089

[61] Salur, E., Acarer, M. & Şavkliyildiz, İ. Improving mechanical properties of nano-sized TiC particle reinforced AA7075 Al alloy composites produced by ball milling and hot pressing. *Mater. Today Commun.* **27**, 102202 (2021). 10.1016/j.mtcomm.2021.102202

[62] Hamid, F. S., El-Nikhaily, A., Abd Ellatif, H. & Elkady, O. A. Morphology and mechanical properties of Al- TiC nanocomposite processed via ball milling technique. *Int. J. Mater. Technol. Innov.* **1**, 18–29 (2021).

[63] Li, L. *et al.* A simple approach to obtaining enhanced mechanical properties of graphene/copper composites with heterogeneous grain structures. *Mater. Sci. Eng. A* **832**, 142438 (2022). 10.1016/j.msea.2021.142438

[64] Mothopeng, N., Maledi, N., Maminza, M. & Chikosha, S. A comparative corrosion study of titanium strips produced by wrought and direct powder rolling processes. *IOP Conf. Ser. Mater. Sci. Eng.* **430**, 012041 (2018). 10.1088/1757-899X/430/1/012041

